# Uncovering cancer gene regulation by accurate regulatory network inference from uninformative data

**DOI:** 10.1038/s41540-020-00154-6

**Published:** 2020-11-09

**Authors:** Deniz Seçilmiş, Thomas Hillerton, Daniel Morgan, Andreas Tjärnberg, Sven Nelander, Torbjörn E. M. Nordling, Erik L. L. Sonnhammer

**Affiliations:** 1 Department of Biochemistry and Biophysics, Stockholm University, Science for Life Laboratory, Box 1031, 17121 Solna, Sweden; 2 Center for Developmental Genetics, New York University, New York, NY USA; 3 Science for Life Laboratory, Department of Immunology, Genetics and Pathology, Uppsala University, Uppsala, Sweden; 4 Department of Mechanical Engineering, National Cheng Kung University, Tainan, Taiwan

**Keywords:** Regulatory networks, Cancer, Reverse engineering

## Abstract

The interactions among the components of a living cell that constitute the gene regulatory network (GRN) can be inferred from perturbation-based gene expression data. Such networks are useful for providing mechanistic insights of a biological system. In order to explore the feasibility and quality of GRN inference at a large scale, we used the L1000 data where ~1000 genes have been perturbed and their expression levels have been quantified in 9 cancer cell lines. We found that these datasets have a very low signal-to-noise ratio (SNR) level causing them to be too uninformative to infer accurate GRNs. We developed a gene reduction pipeline in which we eliminate uninformative genes from the system using a selection criterion based on SNR, until reaching an informative subset. The results show that our pipeline can identify an informative subset in an overall uninformative dataset, allowing inference of accurate subset GRNs. The accurate GRNs were functionally characterized and potential novel cancer-related regulatory interactions were identified.

## Introduction

Living organisms are orchestrated by the biochemical reactions that occur as a result of the interactions between biomolecules. For that reason, understanding the biochemical, physiological, and pathological processes from a gene regulation perspective is of importance. These processes in a living organism on a gene level can be represented via a gene regulatory network (GRN) that can be inferred from perturbation-based gene expression data, e.g., where each gene in the system is knocked down in a separate experiment. Such GRNs are useful for providing mechanistic insights of a biological system. Accurately performed inference on cancer data might propose alternative treatments since novel gene regulatory links carry the potential to identify new drug targets.

Several GRN inference methods have been proposed including least squares, LASSO^1,2^, Robust Network Inference algorithm (RNI)^3^, Context Likelihood of Relatedness (CLR)^4^, Genie3^5^, and Inferelator^6^. However, the inference of the mentioned GRNs from the biological data has severe limitations, among which the most crucial one is noise of the dataset which negatively affects the performance of any inference method. It has been shown that even the best performing inference methods tend to exhibit very poor accuracy if the signal-to-noise ratio (SNR) is low^7^, and that real datasets have low SNR^8^. Previous work has been done in order to propose solutions for this problem in GRN inference, mostly exploiting stability during bootstrapping as a means to reduce the effect of noise. For instance, a nested bootstrapping algorithm has been proposed to control the false discovery rate (FDR) in GRN inference of noisy datasets, in order to improve the accuracy of the applied method^8^. However, these solutions are applied directly to inferred GRNs of noisy datasets, and if the dataset as a whole is not informative then the improvement may be limited.

In this study, in an attempt to overcome the challenge of experimental noise, a subset-selection pipeline was developed where the main aim is to improve the SNR of the dataset by permanently removing the least informative genes and their experiments from the system until reaching an informative subset. The novelty of this study lies in the collection of the most informative genes in a dataset before the GRN inference is performed, and reaching a subset allowing the inference of the accurate GRNs.

## Results

Previous studies have shown that accurate GRN inference relies mainly on the SNR of the datasets^7–10^. For this reason we developed a subset-selection pipeline to improve the SNR level of the system.

**Fig. 1:**
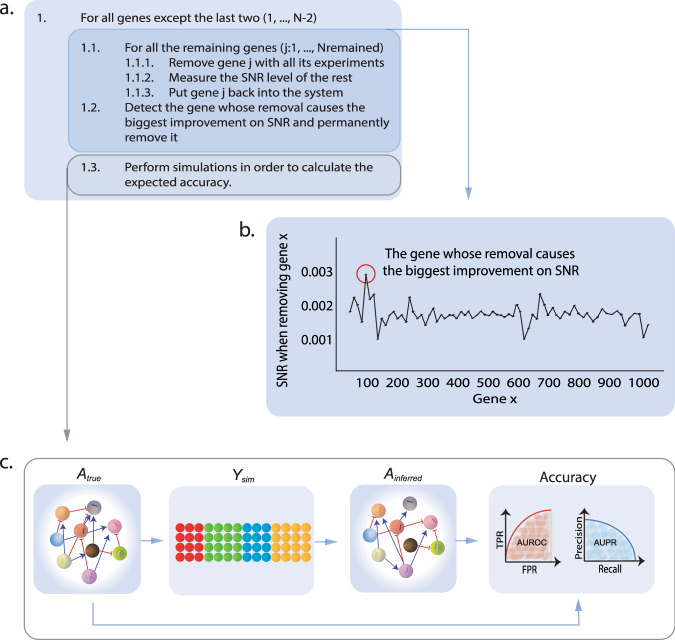
Workflow of the subset-selection algorithm. **a** The subset-selection algorithm, where each gene is removed together with its knockdown experiments, and SNR of the remaining dataset is measured. The gene is then put back and the procedure is repeated for all genes in the dataset. **b** The inner part of the algorithm showing the changes in SNR after each removal and the detection of the gene whose removal increases SNR the most and therefore will be permanently removed. **c** The simulation step applied for the calculation of the expected accuracy of the GRN inference, where *A*_true_ refers to the true GRN that can either be fully synthetic or estimated from the real data, *Y*_sim_ denotes the generated expression matrix from *A*_true_, *A*_inferred_ is the inferred GRN, while the accuracy was evaluated in terms of the area under the ROC and precision-recall curves.

In order to identify the genes lowering the system’s SNR level, the pipeline temporarily removes each gene from the dataset, and the SNR of the remaining subset is measured followed by the return of the removed gene into the dataset. After this is done for all genes, the gene whose removal caused the largest SNR gain is permanently removed from the system. This procedure is repeated for each reduced subset until only two genes remain (Fig. 1a, b).

**Fig. 2:**
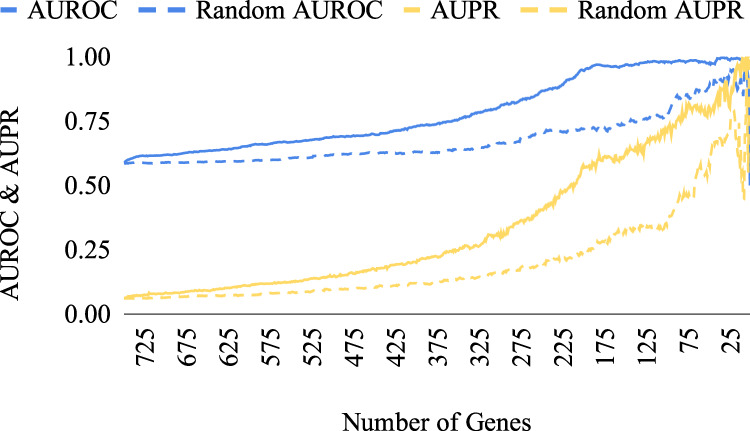
Performance of the subset-selection algorithm on the 750-gene GeneSPIDER synthetic dataset. The performance in terms of AUROC and AUPR of the subset-selection algorithm is shown, compared to the performance of random gene removal. The *x*-axis represents the remaining subset size.

**Fig. 3:**
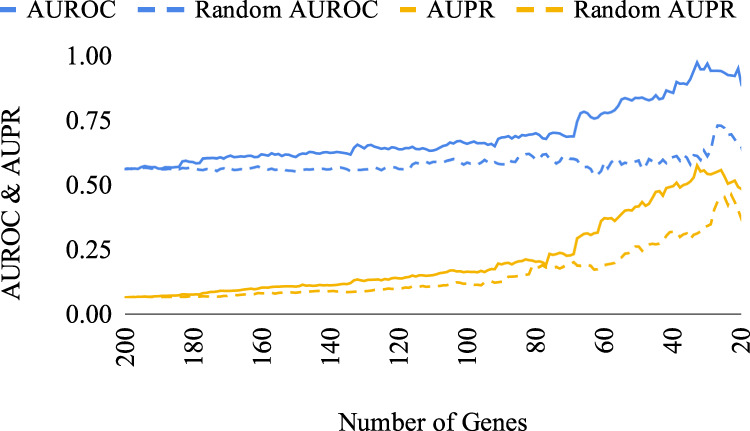
Performance of the gene reduction algorithm on the 200-gene GeneNetWeaver synthetic dataset. The performance in terms of AUROC and AUPR of the subset-selection algorithm is shown, compared to the performance of random gene removal. The *x*-axis represents the remaining subset size.

The reduction algorithm was applied to data from generated true GRNs of size 250–1000 genes from GeneSPIDER^7^, and a 200-gene GRN and dataset from GeneNetWeaver (GNW)^11^ for assessing the performance of the pipeline. The genes were also removed randomly for comparison. Figure 2 exhibits the performances of the reduction algorithm and of random removal in terms of the area under the receiver operating characteristic and precision-recall curves (AUROC and AUPR, respectively) for the size of 750 genes. Accuracy results from other sizes are displayed in Supplementary Figs. 1–3. Figure 3 shows the performance of the reduction algorithm on the GNW data in comparison with random removal. Each AUROC and AUPR point in Figs 2 and 3 were obtained by GRN inference with least squares with cut-off (LSCO)^12^ from the reduced dataset, as described in the “GRN inference methods” subheading in the Methods section.

The reduction algorithm improves accuracy and SNR considerably better than random selection. The reason that random subset selection has a positive effect on these measures is that smaller subsets have fewer degrees of freedom and are therefore easier to predict and have higher SNR. To assess the statistical significance of the difference between the performances of the reduction algorithm and random removal, the paired Wilcoxon test was applied to the SNR, AUROC, and AUPR levels obtained from both removal strategies. The use of a nonparametric test was due to not meeting the normality assumption in any of the variables according to the Shapiro–Wilk test. For all measures from all dataset sizes except one (AUPR of 250 genes), the reduction algorithm was statistically significantly different (*p* < 3.1e−05) from random removal (Supplementary Table 1).

When starting with 750 genes in the GeneSPIDER data, AUROC reached a plateau at almost the perfect level and AUPR made a sharp increase when ~200 genes remained in the dataset (Fig. 2). 500- and 1000-gene datasets followed a similar trend for AUROC in terms of reaching a plateau, and a constant increase until the very end was observed for the AUPR values with the 500-gene dataset performing considerably better than the 100-gene dataset (Supplementary Figs. 2 and 3). On the other hand, for the 250-gene dataset a constant increase in both AUROC and AUPR values was observed, which is probably due to the small starting size (Supplementary Fig. 1).

For the 200-gene GNW data, a marked increase in both AUROC and AUPR values occurred when ~65 genes remained, and the AUROC level reached almost 1 when ~30 genes remained (Fig. 3).

We also investigated a simpler approach of selecting genes based on their entropy (Supplementary Figs 51–54). For all starting sizes, this method did not outperform random selection in terms of AUROC. For starting sizes 500 and 750 genes it also did not improve compared to random selection in AUPR, but for 250 and 1000 genes some improvement was observed, yet at modest AUPR levels. In conclusion, the entropy-based subset-selection method generally performed poorly and cannot be recommended.

In the analyses and inferences of the selected L1000 datasets, the reduction pipeline was run on the datasets and the SNR level of subsets of each cell line was measured. Simulation and benchmarking of expected inference accuracy at different SNR levels using in silico data were performed. Finally, GRNs were inferred from the most informative subset of each cell line and functional analysis and classification were made for these accurate subset GRNs.

### Benchmarking the reduction pipeline

**Fig. 4:**
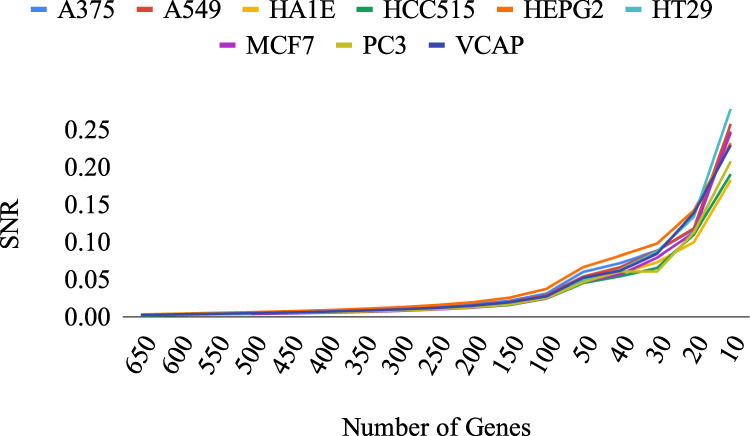
The SNR level of SNR-enriched subsets of the nine selected L1000 cell lines. The *x*-axis represents the subset size, the *y*-axis denotes the SNR, and each curve represents a cell line.

The subset-selection pipeline was applied to all L1000 cell lines. It progressively removes the gene whose exclusion improves SNR the most, continually increasing the SNR of the dataset. In Fig. 4, the gradual increase of the SNR levels reflecting the informativeness of the remaining subsets is shown.

In order to determine the most suitable inference method for network generation and GRN inference, both LSCO and LASSO were benchmarked on different subset sizes and SNR levels. The results from the A375 cell line are shown in Fig. 5, and from all cell lines in Supplementary Figs 22–39.

**Fig. 5:**
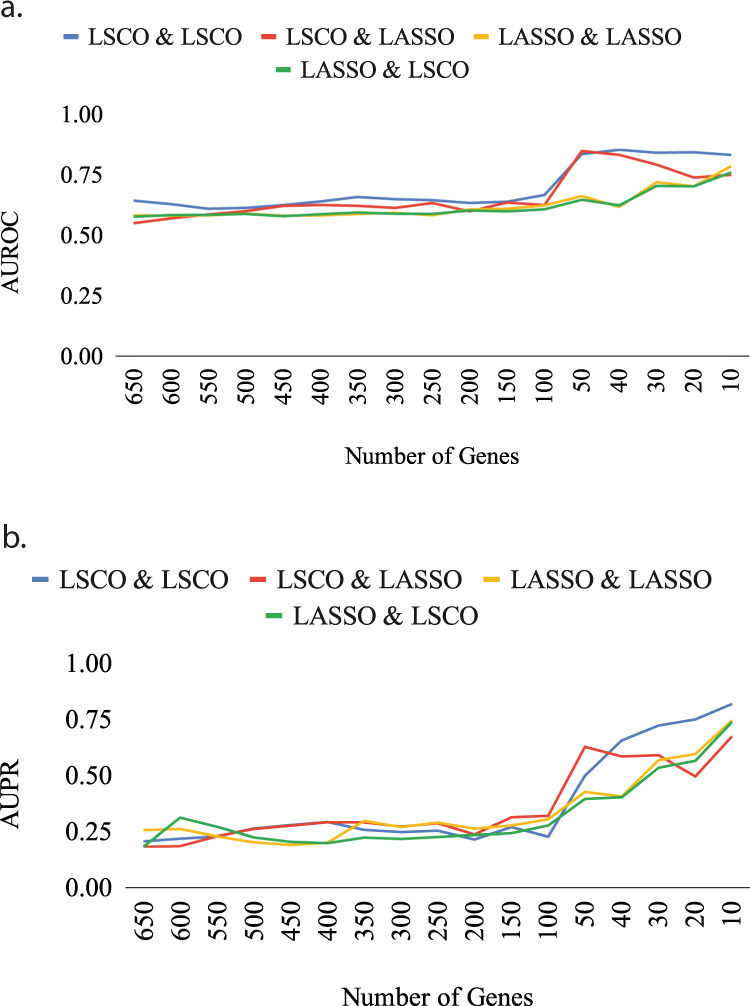
Evaluation of methods for benchmarking GRN inference accuracy with simulation. The evaluation was made for the subsets of the A375 cell line in terms of AUROC (**a**) and AUPR (**b**). The legend shows the algorithm used to generate the true GRN for benchmarking, and the algorithm used to infer the GRNs from the simulated data as the first and second labels. For example, ‘LSCO & LASSO’ denotes that the true GRN was generated with LSCO and the GRNs were inferred with LASSO.

It can be observed from the benchmarking of the two inference methods on the subsets of A375 cell line (Fig. 5) that a radical increase in both AUROC and AUPR levels starts at the 50-gene subset, and that LSCO generally outperforms LASSO at both true GRN generation and GRN inference. Therefore, the LSCO method was chosen for both the true GRN generation and the GRN inference for its high accuracy and computational efficiency.

**Fig. 6:**
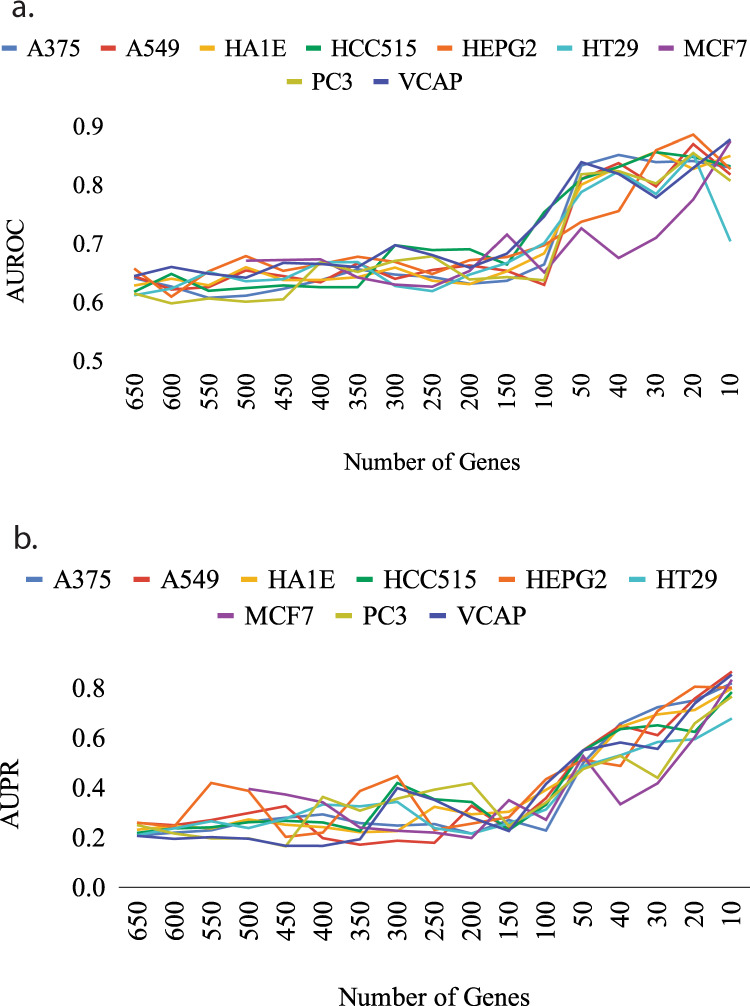
Expected accuracy of subset GRNs. The accuracy was derived from simulations using LSCO and measured as AUROC (**a**) and AUPR (**b**) on the nine L1000 cell lines. The *x*-axis represents the subset size, the *y*-axis the AUROC and AUPR values, and each curve represents a cell line.

The accuracy for subsets using LSCO sharply improves at around 50 genes (Fig. 6). For this reason, the inference of the “accurate GRNs” was performed on the 50-gene subsets of all cell lines.

### Inference of accurate subset GRNs from L1000 subsets

**Fig. 7:**
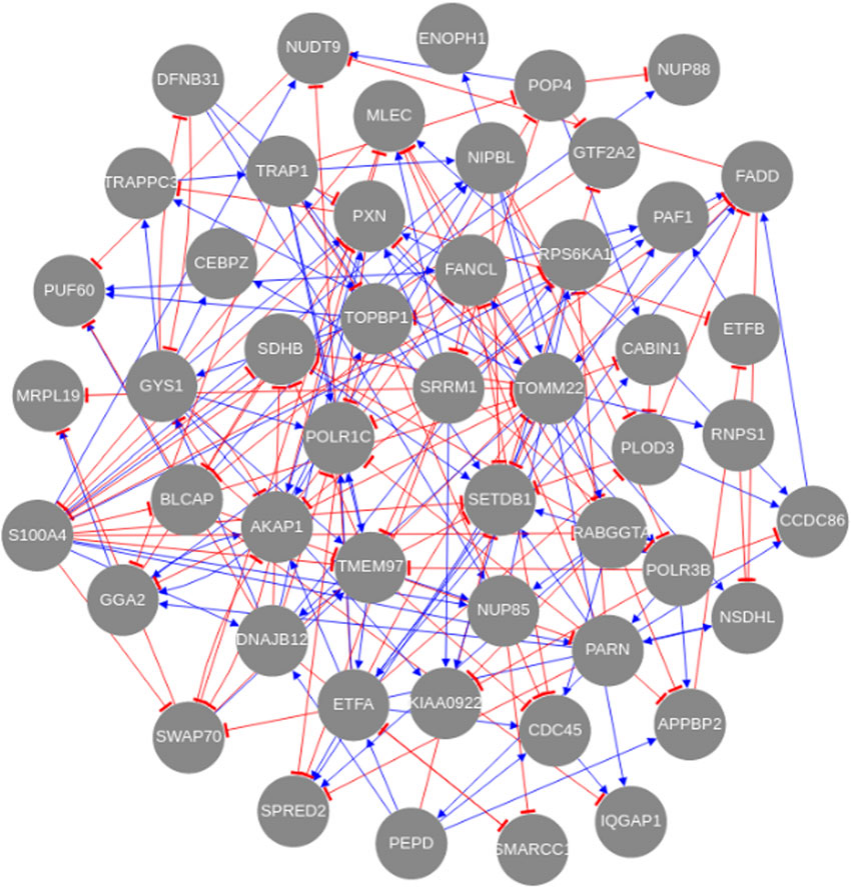
The accurate GRN of the HT29 colon cancer cell line. The nodes demonstrate the informative genes of this L1000 cell line, and blue and red edges represent the regulatory interactions which are either activation or suppression, respectively.

After establishing that the 50 most informative genes result in accurate GRNs, we next inferred such GRNs by applying LSCO to these subsets in each of nine L1000 cell lines. An average sparsity of about 3–5 links per gene is considered biologically plausible^3,13,14^. In order to get close to this, we selected GRNs with a sparsity ranging from 2 to 5 links per gene. Among these, the one whose number of links matches best with the number from the simulation resulting in the most optimal accuracy was selected, and called “the accurate GRN”. The accurate subset GRN of the HT29 cell line is shown in Fig. 7, and the accurate subset GRNs of all nine L1000 cancer cell lines can be found in Supplementary Figs 40–48.

The SDHB gene is a tumor suppressor gene, and has previously been shown to be downregulated in colorectal cancer^15^. However, to our knowledge, possible activators for treatment have not yet been proposed. In our inferred subset GRN for the HT29 cell line (Fig. 7), several genes including DNAJB12, TOMM22, TMEM97, S100A4, RABGGTA, and BLCAP suppress SDHB, suggesting possible mechanisms of SDHB suppression. A database search of these genes was performed in the TRRUST^16^ and RegNetwork^17^ databases but none of these genes was found to regulate SDHB. We also searched the network databases FunCoup^18^ (links with >0.80 confidence), Pathway Commons^19^, and GeneMania^20^ and found support for a link between SDHB and TOMM22 in both FunCoup (confidence 0.997) and GeneMania, and between SDHB and DNAJB12 in GeneMania. We therefore propose that the inferred regulators of SDHB could be valid potential regulatory targets for colorectal cancer treatment.

The inferred subset GRN from the PC3 prostate cancer cell line (Supplementary Fig. 47) suggests several genes as suppressors of SDHB, such as PNKB, PWP1, PNP, HADH, HTATSF1, and BAZ1B, among which PWP1, HTATSF1, and BAZ1B are transcription regulators. BAZB1 is a kinase, and HTATSF1 has RNA-binding activity. SRRM1 activates SDHB in the subset GRN. Again, these relations are not present in the TRRUST and RegNetwork databases. We, however, found that SDHB is linked to PNP and HADH in the FunCoup (confidence = 0.827 and 0.999) and GeneMania networks. The relation between SDHB and HADH also appears in Pathway Commons, and GeneMania links SDHB to SRRM1. These network and pathway connections provide support for the validity of these novel regulatory mechanisms for prostate cancer treatment.

The inferred subset GRN from the MCF7 breast cancer cell line (Supplementary Fig. 46) highlights that the GM2A and PRKACA genes are activated and suppressed by several genes. Overexpression of GM2A in breast cancer has previously been shown^21^, where one of the investigated cell lines was MCF7. In the inferred GRN, GM2A is activated by NOL3, AKT1, KIAA0196, and ACOT9, and suppressed by LIPA, PWP1, JMJD6, and CCDC86, among which PWP1 and JMJD6 are transcription regulators. The JMJD6 protein has RNA binding and histone demethylase activity. Although these interactions with GM2A are not present in the TRRUST and RegNetwork databases, indirect evidence of their validity was found in RegNetwork where GM2A and its identified regulator NOL3 are regulated by two third-party genes, TFAP2A (*q* = 0.0007) that is a DNA-binding transcription regulator and an enzyme, and JUN (*q* = 0.0006), a DNA-binding transcription regulator and oncogene. The likelihood of finding a common regulator by chance was calculated as the square of the total number of targets of each common regulator divided by the total number of protein-coding genes, followed by Bonferroni correction of these probabilities and excluding cases above 0.05. In addition to this, the presence of a link between GM2A and LIPA was found in GeneMania. This adds support to the validity of our identified interactions as potential mechanisms in breast cancer.

Another example is the PRKACA gene, an oncogene that has been linked to breast cancer^22^. The inferred GRN indicates that it is suppressed by NUP85 and IST1H2B, and activated by CHAC1, PWP1, PP2R5E, NOL3, and PNP, of which PWP1, as stated above, is a transcription regulator. Searching the TRRUST and RegNetwork databases did not verify any of our predicted PRKACA interactions, yet the connection between PRKACA and NOL3 was indirectly supported in RegNetwork by a third gene, E2F1, a DNA-binding transcription regulator, that regulates both (*q* = 0.02). Further evidence was found in that FunCoup (confidence 0.991), Pathway Commons, and GeneMania contain a link between PRKACA and NUP85. These observations support the validity of our proposed regulatory mechanisms for breast cancer treatment.

### Functional analysis of the accurate subset GRNs

In order to characterize and validate the accurate subset GRNs, we analyzed them for pathway enrichment, overlap with a functional association network database, and protein class.

The pathway enrichment analysis in this study was performed via PathwAX wherein the genes of the accurate subnetworks were tested for significant association to KEGG pathways^23^ with network crosstalk enrichment analysis. The results are in Supplementary Table 6. We note that cancer-related pathways such as ‘cell cycle’, ‘Oxidative phosphorylation’, and ‘Protein processing’ were significantly enriched in several of the cell lines. Among other recurring pathways we note ‘Alzheimer’s disease’, ‘Parkinson’s disease’, and ‘Huntington’s disease’, which have been linked mechanistically to cancer^24–26^.

The pathway ‘Peroxisome’ was significantly enriched for the subset GRN of the prostate cancer cell line PC3. The peroxisome has previously been linked to prostate cancer^27–30^, and PC3 was one of the investigated cell lines in the study by Mueller et al.^27^. The subset is thus highly relevant for the connection between prostate cancer and the peroxisome. CAT, a member of Peroxisome pathway in the antioxidant system, is in the subset GRN activated by CEBPZ, TMCO1, CLASRP, and PPP2R3A. Of these, CEBPZ is a transcriptional regulator, yet its regulatory effect on the expression of CAT is not known.

The inferred GRNs were further compared to the FunCoup functional association network database^18^ (Table 1). In general, relatively few of the inferred regulatory links were found in FunCoup, which is perhaps not surprising given that FunCoup contains undirected functional links that do not represent regulatory interactions. However, the overlap was significant (*p* < 0.05) in five cell lines with a hypergeometric test. One of these, MCF7, matched no significant pathways above, and PC3 only one pathway, indicating that these GRNs may represent unknown mechanisms. In order to investigate whether the significant match was due to the success of the subset-selection algorithm identifying an informative subset for accurate GRN inference or not, we also inferred GRNs from the full-size datasets of the L1000 cell lines and compared the inferred GRNs with a sparsity of ~3 links per node on average to the related FunCoup networks and found that no overlap was significant (Supplementary Table 5).

**Table 1:** The comparison of the accurate subset GRN of each cell line to the related FunCoup network of the same genes.

Cell line	Overlap	Links in FunCoup	Links in the inferred GRN	*p*-Value
A375	6	36	84	0.001*
A549	4	106	130	0.83
HA1E	6	90	96	0.14
HCC515	6	74	104	0.09
HEPG2	7	33	221	0.02*
HT29	13	97	197	0.04*
MCF7	19	79	125	5e−09*
PC3	10	77	91	0.0004*
VCAP	10	88	170	0.08

* Marks statistical significance with *p* < 0.05.

**Fig. 8:**
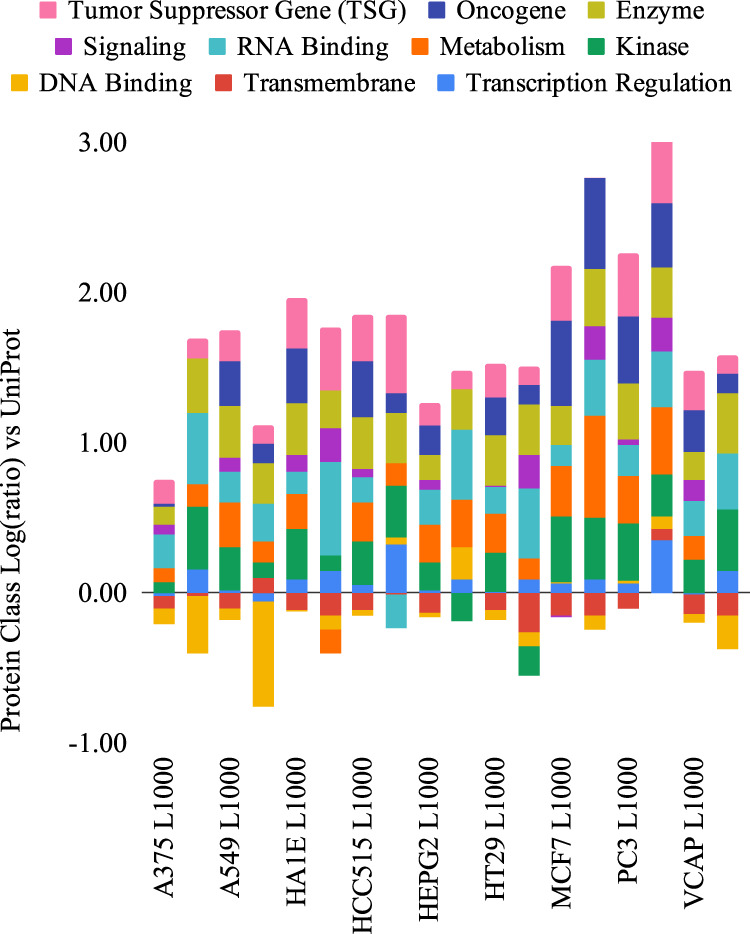
Protein class enrichment of each cell line and their 50-gene subsets. The *x*-axis shows the cell line, and the *y*-axis shows the class enrichment relative to UniProt fractions as log(ratio).

Protein class analyses were performed for the genes of the selected subsets in terms of the activity of their proteins. In order to achieve this, the human proteome was downloaded from UniProt^31^, including the keywords transcription regulation, transmembrane, DNA binding, kinase, metabolism, RNA-binding, enzyme (from molecular function), and signaling. Additional data were downloaded from COSMIC^32^ from which oncogenes and tumor suppressor genes were obtained. The genes belonging to each selected cell line were assigned a protein class according to this scheme (Fig. 8).

Most of these classes tend to be enriched in L1000 gene sets relative to UniProt, particularly kinase, metabolism, enzyme, oncogene, and tumor suppressor gene. Mainly transmembrane and DNA binding tend to be depleted. The highest enrichments in subsets relative to their original L1000 set were observed for A375 (kinase, enzyme, RNA binding), MCF7 (signaling, RNA binding, metabolism), PC3 (transcription regulation, signaling, RNA binding), and HCC515 (transcription regulation). These cell lines thus underwent a further enrichment of regulatory functions during the subset selection. A few cases of further depletion were also observed, for instance of DNA binding in A375 and A549.

### Knockdown effect on the target

Since a successful knockdown of the target gene is important, the knockdown effects on the targets were investigated and are shown in Fig. 9. Volcano plots of the knockdown effects are provided in the supplementary material (Supplementary Figs. 4–21). Significance analyses of the targeted genes show that a majority of the targets were successfully downregulated. However, in addition to the nonsignificant changes in their expressions, a portion of the targets was observed to be significantly upregulated. In A375, A549, HCC515, HEPG2, and HT29, 6–10% of the genes were significantly upregulated, while this proportion increases up to 20–26% in HA1E, MCF7, PC3, and VCAP cell lines.

**Fig. 9:**
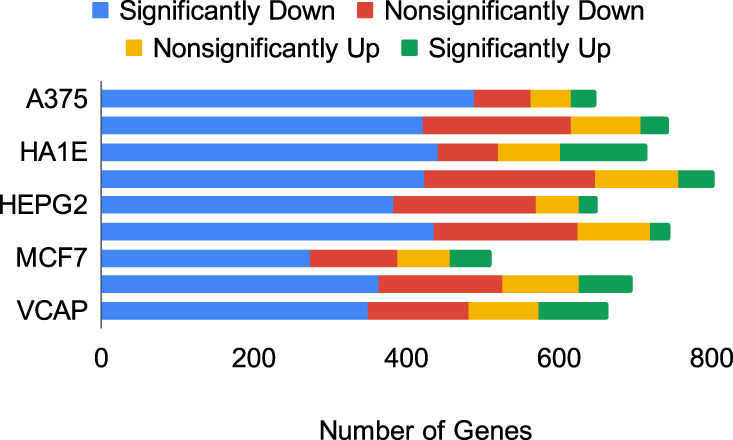
Knockdown effect on target. Significant and nonsignificant up- and downregulation of the shRNA target genes in the studied L1000 cell lines.

## Discussion

Uninformative data have previously been shown to negatively affect the performance of GRN inference methods, leading to poor accuracy. In order to overcome this problem, a progressive subset-selection method is proposed, removing the genes that are the most affected by noise. This approach proved to capture the most informative and therefore the most accurately inferrable subsets, which can be considered a major advance in the GRN inference area since the high noise problem is a general issue for all real datasets.

The subset-selection algorithm was validated by synthetic data and true GRNs. The performance of the algorithm was shown to significantly outperform random removal of genes. We applied the algorithm to L1000 datasets of nine selected cancer cell lines and observed that we could increase SNR to a level that permits accurate GRN inference.

We examined whether the experimental L1000 perturbations were successful via nonparametric significance analysis of the targeted genes’ expression and visualized them by volcano plots. A majority of the target genes were significantly knocked down compared to their controls. However, also many significantly upregulated genes were observed, as well as nonsignificantly up- and downregulated ones. One possible reason for this from the biological perspective is that these genes are highly important for the system, and their expression is immediately restored by feedback mechanisms in the cell. If the reaction is very strong, this could lead to temporal overcompensation and an observed increase in expression. This could be revealed by a time series of measurements, but unfortunately the L1000 shRNA perturbations are limited to the 96 h time point.

In the benchmarking of the real data, using in silico datasets where the properties of the real datasets are mimicked, a significant improvement in LSCO’s inference accuracy in terms of AUROC and AUPR was observed when increasing the SNR level of the dataset as the subset becomes smaller. The improvement was stronger and came earlier for LSCO compared to LASSO, especially when used for inferring a true GRN for simulations. Given this variability, it is possible that combinations of other inference algorithms might perform even better.

The benchmarking of different subset sizes in different cell lines with LSCO indicated that in order to infer accurate GRNs, the minimum SNR level of the dataset should be at least ~0.05. For the L1000 datasets, this means losing more than 90% of the initial data with the proposed subset-selection algorithm. However, in a situation where the majority of the genes have noisy measurements, it becomes unbeneficial to include them in the system. In GRN inference where the aim is to reliably discover regulatory interactions, including noisy data that lowers the informativeness of the dataset is unacceptable. On the other hand, the possible removal of important genes such as oncogenes may obscure their potential interactions with other genes, and cause their effect in cancer to remain unknown. In an attempt to minimize this risk, such genes can be kept. However, then more genes must be sacrificed in order to achieve the desired SNR for the inference to be accurate. This would result in a smaller subset, which may be a greater drawback.

The time complexity of the subset-selection algorithm is in principle O(n^2^) for n genes. However, due to the currently used SNR calculation at each step this is further increased to O(n^3^) because it relies on singular value decomposition. This time complexity means that while it is fast up to a few hundred genes, for datasets of around 1000 genes several days of computation might be needed to complete the subset selection.

One result of this study is accurate subset GRNs for nine cancer cell lines. These include a large number of new regulatory interactions among transcriptional regulators and oncogenes, and suggest new potential therapeutic targets. We have focussed on a few cases of cancer relevance that would deserve experimental follow-up and validation.

## Methods

### Calculation of the SNR

The smallest value from the singular value decomposition of the gene expression matrix is considered to be the signal, and the variance multiplied by a degrees-of-freedom-dependent chi-square constant is considered to be the noise in this study. The ratio between the signal and noise is shown in Eq. (1).

1 $${\mathrm{SNR}} \equiv \frac{{\sigma _N\left( Y \right)}}{{\sqrt {\chi ^{ - 2}\left( {1 - \alpha ,NM} \right)\lambda } }}$$

where σ_*N*_(*Y*) denotes the smallest value from the singular values of the expression matrix *Y*, $\sqrt {\chi ^{ - 2}\left( {1 - \alpha ,NM} \right)}$ the inverse chi-square distribution with 1 − *α* confidence level and *NM* degrees of freedom (*N* genes, *M* samples), and *λ* the variance of the noise.

### Datasets

The pipeline was applied to selected datasets from the LINCS L1000 data^33^. However, for the calculation of the expected accuracy of the inference and the validation of the existing links in the corresponding GRN, in silico networks and perturbation datasets with properties similar to the real datasets were generated ranging from the full size of the dataset to smaller but more informative subset sizes. Supplementary Figs 22–39 show all the benchmark results using either LSCO or LASSO for inference of a true GRN to generate simulated data as well as inference of GRNs from simulated data, in terms of AUROC and AUPR measurements for the subsets of the nine L1000 cell lines.

### Synthetic GRNs and datasets

In this study, we used two different in silico GRN and data generation tools, namely GeneSPIDER^7^, and GeneNetWeaver^11^ (GNW). In the synthetic data, the most important property is considered to be the SNR due to the basis of the proposed subset-selection algorithm. For this reason, we set the SNR levels of the GeneSPIDER datasets to 0.001 in accordance with the SNR levels of the L1000 datasets (Table 2). For the GeneSPIDER datasets, this was possible as the tool itself allows this property to be set by the user. However, in GNW, this was not an option. On the other hand, by using their default settings for the noise parameters (0.05 coefficient for stochastic, and 0.025 standard deviation for the Gaussian noise), we were able to obtain a final dataset with 0.0079 SNR, which is reasonably close to the SNR levels of the L1000 and GeneSPIDER datasets.

**Table 2:** The main properties of the L1000 datasets that are used in the subset-selection pipeline.

Cell line	Tissue	Size	SNR
A375	Skin	649 × 1879	0.0020
A549	Lung	744 × 2016	0.0017
HA1E	Embryonic kidney	716 × 2092	0.0017
HCC515	Lung	803 × 2390	0.0010
HEPG2	Liver	650 × 2129	0.0030
HT29	Colon	746 × 2901	0.0012
MFC7	Breast	512 × 1282	0.0030
PC3	Prostate	697 × 1670	0.0023
VCAP	Prostate	664 × 1717	0.0021

The sizes of the datasets are represented by (genes × experiments).

In order to explore the effectiveness of the subset-selection algorithm for various SNR values, we also generated two different datasets from the 250-gene GeneSPIDER network with SNR of 0.01 and 0.0001 which are 10-times higher and lower, respectively, than the biologically realistic SNR values (~10^−3^) (Table 2). The accuracy of the subset selection on these datasets can be found in Supplementary Figs 49–50.

### Generation via GeneSPIDER

To assess the performance accuracy of the algorithm, synthetic GRNs in various sizes (250, 500, 750, and 1000 gene), and datasets from these GRNs with three replicates per gene and SNR of 0.001 were generated with the GeneSPIDER Network.m and Dataset.m tools. The true GRNs were generated in scale-free topology allowing three links per gene on average. Random Gaussian noise was generated with a standard deviation calculated specifically to meet the requirement for the desired SNR level, and then added to the noise-free gene expression matrix generated from the true GRN (Eqs. 1–2).

For real data, “true GRNs” were generated by first inferring GRNs at several sparsities with the least-squares cut-off (LSCO) method, and then selecting the most sparse GRN with at least 2 links per node on average. The preference of this method was made based on its computational efficiency on large scale datasets and competitive accuracy shown in the small benchmark whose results are provided in Fig. 5 for A375 cell line and in the supplementary material for all nine cell lines (Supplementary Figs. 22–39). Second, these inferred networks were used as the true GRNs for the generation of the in silico datasets, which was performed by involving the perturbation design matrices (***P***) of the real datasets in order to directly reflect the number of replicates into the simulation, as well as adding random noise calculated based on the SNR levels of each dataset and their subsets.

The linear model for the expression matrix generation is shown in Eq. (2).

2 $$\displaystyle {Y_{{\mathrm{Generated}}}} =\displaystyle - \left( {A_{{\mathrm{True}}}} \right)^{ - 1} \times P_{{\mathrm{Real}}} + E_{{\mathrm{Generated}}}$$

In Eq. (2), *A*_True_ is the network matrix which is inferred from the real dataset with the LSCO method and then stabilized. *P*_Real_ indicates the original perturbation matrix of the real dataset or its subset, *E*_Generated_ the random noise matrix generated based on the SNR level of the real dataset that the simulations are performed for, and *Y*_Generated_ the generated expression matrix. *Y*_Generated_ and *P*_Real_ are used to infer a GRN, which is then compared to *A*_True_ for calculating the expected accuracy of the inference of the real dataset. The simulation procedure is visualized in Fig. 1c.

### Generation via GeneNetWeaver

To assess the performance of the proposed subset-selection pipeline on another synthetic data coming from a different tool, an additional true GRN of 200 genes was extracted from the *E. coli* network that is available in GNW, and three datasets from this “true” GRN were generated that were afterward used as the biological replicates in one merged dataset of size 200-by-600. The 200-gene network was extracted from the full-size *E. coli* network by requesting at least 100 regulators from random vertices, and neighbor selection was made by the GNW setting “random among top 50%”. A kinetic model was then generated by keeping all autoregulators, which was used to generate datasets. Three knockdown datasets from this kinetic model were generated from a combination of ordinary differential equations and stochastic models. We avoided the normalization step at this moment, and saved it for the merged dataset. We further calculated the log2 fold changes for each dataset as the logarithm base 2 of the ratio between each gene’s expression and the control. Some of the control values happened to be zeros, which would cause infinite values for the fold changes as matlab assigns these for the division by zero. To avoid infinite values in the fold changes, we added an arbitrary value of 0.001 to all controls. We then set the infinite log2 fold change values to zeros as matlab assigns infinite values to log_2_(0). After this was done for all three datasets, we merged them into one single data matrix, and then normalized each gene over its three replicates by subtracting the mean and dividing by the standard deviation.

### Reduction of true GRN during subset selection

For synthetic data, the true GRN of the dataset is reduced iteratively in accordance with the removal of the same gene from the dataset. However, due to the lack of a true GRN in the case of real data, a different approach is applied here. At regular subset intervals (*N* = 50), a simulation step is included in this pipeline which generates a subset GRN and data with the same SNR as the real data subset, which is used to estimate the expected accuracy of a GRN inferred for the subset (Fig. 1c).

### L1000 datasets

L1000 is a collection of perturbation datasets produced by the namesake platform, including shRNA (short-hairpin RNA), small molecule and protein perturbations performed on 77 cancer cell lines, gathered from the measurements on various time points such as 6, 12, 24, 72, 96, 120, and 144 h. The Q2Norm Level 3 L1000 data were downloaded from https://www.ncbi.nlm.nih.gov/geo/ with the accession GSE92742 including all the mentioned types of perturbations, time points, biological and technical replicates of the measured and inferred genes. Level 3 was used because it is the only provided normalized data that does not collapse replicates. Only directly measured expression levels were used, not inferred ones. In this study, only the shRNA perturbations collected at the 96 h time point in eight cell lines (A375, A549, HA1E, HCC515, HEPG2, HT29, MCF7, and PC3) and 120 h of one cell line (VCAP) were considered for inference. The inconsistent number of biological replicates per gene is not considered a problem, hence all biological replicates were kept. In each biological replicate, usually multiple shRNAs are used that target different parts of the same gene in order to mitigate off-target effects. However, sometimes different biological replicates for the same target gene use different sets of shRNAs. To avoid biases we only kept those common among all biological replicates of one target gene.

Fold changes were calculated for GRN inference using the included empty vector controls per cell line. Table 2 describes the dimensions in each of the nine selected cell lines (genes × experiments) as well as their SNR level.

### Performance evaluation

The performance of the subset-selection pipeline was calculated from synthetic true GRNs and datasets. The pipeline was applied by removing the genes not only from the dataset but also from the true GRN to calculate the accuracy of inferred GRNs after every removal and for further assessment. As a baseline for comparison, genes were also removed randomly from the true GRNs and datasets. This enabled the calculation of the statistical significance of the proposed pipeline, which are shown in Supplementary Table 1. To compare SNR-based subset selection to a procedure based on variation, we also applied entropy-based removal on the synthetic data where the gene entropy was calculated as in Zambelli et al.^34^ (Supplementary Figs. 51–54). The limitation of not having known GRNs for the L1000 datasets is overcome by means of synthetic data generated from the “true GRNs” that are generated as described above. Inferred GRNs based on these in silico datasets can then be benchmarked against the “true GRNs”. GRN inference can be performed via a variety of perturbation-based inference methods such as LSCO or LASSO. Both of these methods were applied to each subset.

The decision of the best inference method in each case was followed by the detection of the optimal GRN sparsity providing the best accuracy among all the inferred ones ranging from full to empty. In this case, rather than the application of a certain model selection criterion such as AIC^35^ or BIC^36^, a manual selection was made based on the consideration of a biologically meaningful sparsity level (allowing ~3 links per node)^12,13^ and multiple accuracy measures including true positive rate (TPR, or recall, or sensitivity), false-positive rate (FPR) and precision (in terms of the area under the ROC and PR curves). The reason for selecting a manual sparsity optimization over a criterion taking its place in the literature is due to the fact that all of these model selection criteria point out the best model among all the others. However, it does not guarantee the best model in general. On the contrary, the manual selection in this study with an arbitrary approximation of the outgoing links set to three per gene enables multiple accuracy measures to be taken into account together in the selection of the desired GRN.

### GRN inference from the real datasets

After the detection of the best performing method for each subset of each cell line as well as the GRN with the optimal sparsity level was made, real GRNs from the subsets of the L1000 cell lines could be inferred via the best performing inference methods with an expected level of accuracy calculated in the simulations. This was followed by the identification of the desired GRNs that the simulations suggest.

Once having the “accurate GRN”, the inferred links and their corresponding genes should be evaluated. For this reason, a database search was performed in PathwaX^37^ for pathway enrichment analysis.

In addition to this, functional class analysis was performed in terms of the proteins coded by the genes of interest. The outputs are presented and discussed in the “Results” and “Discussion” sections, respectively.

### GRN inference methods

In this study we used two GRN inference approaches, least-squares cut-off (LSCO)^12^ and LASSO^1,2^, using existing wrappers implemented in the GeneSPIDER Matlab toolbox. The Glmnet implementation of LASSO was used, which applies an L1-regularized penalty for the sparsity of the inferred GRN. For LSCO, the wrapper implements a sparsity cut-off for the inferred least-squares regression coefficients in order to achieve a set of GRNs whose sparsities range from full to empty. Using both methods, 50 GRNs of different sparsity were inferred for each dataset and compared to the true synthetic GRNs to obtain the areas under the ROC and precision-recall curves. Inferred GRNs having ~2–5 links per gene on average were mainly considered for biological database validation.

## Supplementary information

Supplementary Material for “Uncovering cancer gene regulation by accurate regulatory network inference from uninformative data”

## Data Availability

The source code of the subset-selection algorithm, and a small demo can be found at https://bitbucket.org/sonnhammergrni/genespider/src/subset-selection/ as SubsetSelectionSynthetic.m and SubsetSelection.m, respectively.

## Data availability

The synthetic networks and datasets are available at https://bitbucket.org/sonnhammergrni/genespider/downloads/ as .mat files for four different dataset sizes for GeneSPIDER and one size for GNW. The real L1000 datasets are available at https://www.ncbi.nlm.nih.gov/geo/ with the accession GSE92742.
