# Supplementary material for: Uncovering cancer gene regulation by accurate regulatory network inference from uninformative data
Source: NPJ Syst Biol Appl. 2020 Nov 9;6:37. doi: 10.1038/s41540-020-00154-6 (PMC7652823; doi:10.1038/s41540-020-00154-6)
Supplement: Supplementary file 1 — Supplementary Material for “Uncovering cancer gene regulation by accurate regulatory network inference from uninformative data” [file 41540_2020_154_MOESM1_ESM.pdf]

Supplementary Material for "Uncovering cancer gene regulation by accurate regulatory network inference from uninformative data"

Supplementary Table 1. P-values from the paired Wilcoxon test for the difference between the performances of the subset selection algorithm and random removal in different starting sizes. * marks the statistical significance with 95% confidence.

Size	SNR	ROC Curve	PR Curve
**200 (GeneNetWeaver)**	< 2.2e-16	< 2.2e-16	< 2.2e-16
**250 (GeneSPIDER)**	< 2.2e-16*	9.14e-16*	0.79
**500 (GeneSPIDER)**	< 2.2e-16*	< 2.2e-16*	< 2.2e-16*
**750 (GeneSPIDER)**	< 2.2e-16*	< 2.2e-16*	< 2.2e-16*
**1000 (GeneSPIDER)**	< 2.2e-16*	< 2.2e-16*	3.1e-05*

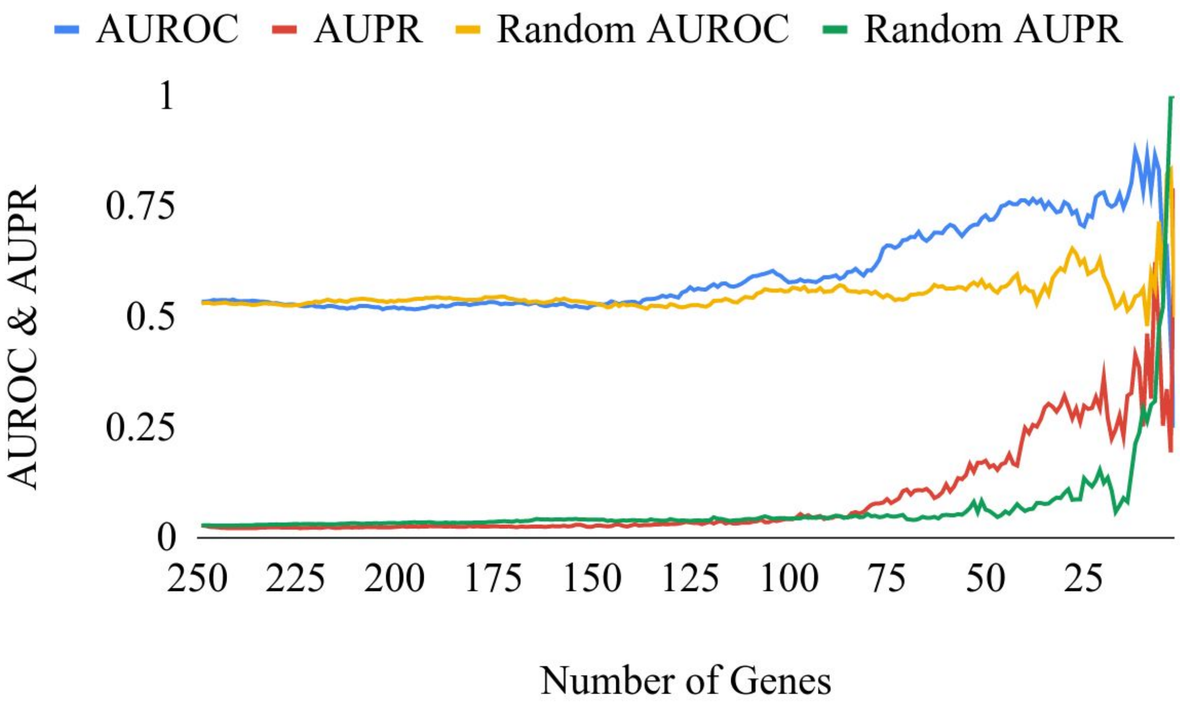

Supplementary Figure 1. Performances of both the gene reduction algorithm and random removal on a 250-gene synthetic dataset. The x-axis represents the subset size, and the y-axis denotes the AUROC and AUPR values.

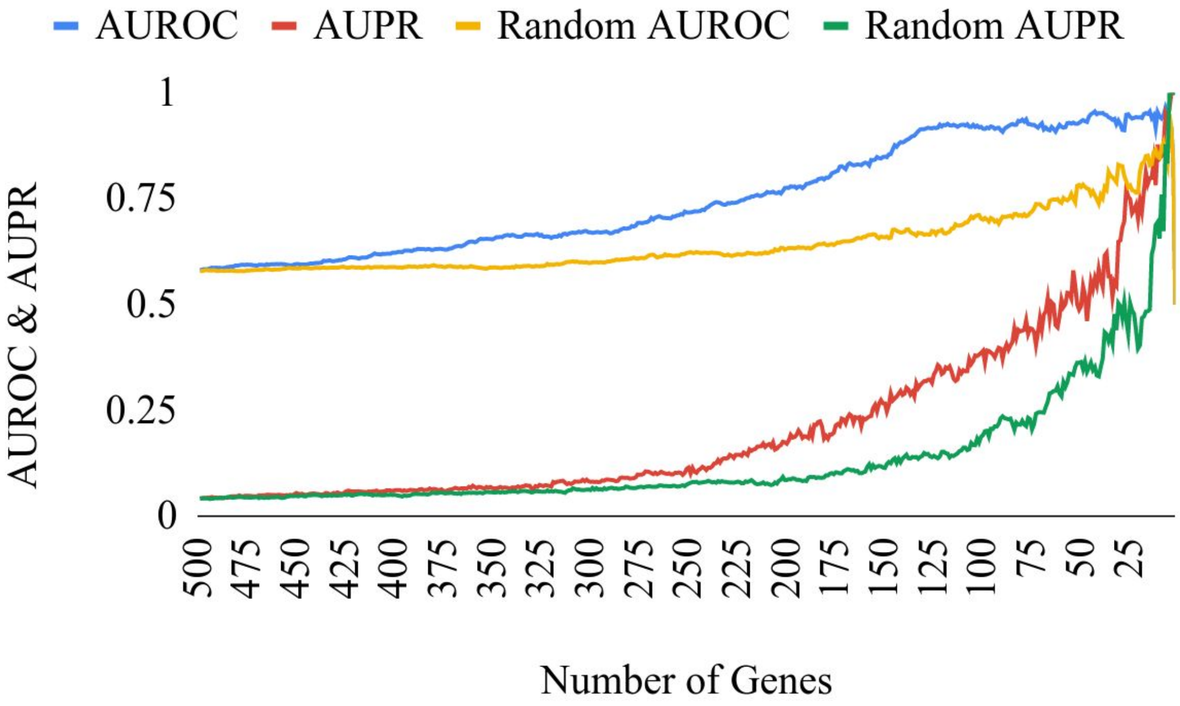

Supplementary Figure 2. Performances of both the gene reduction algorithm and random removal on a 500-gene synthetic dataset. The x-axis represents the subset size, and the y-axis denotes the AUROC and AUPR values.

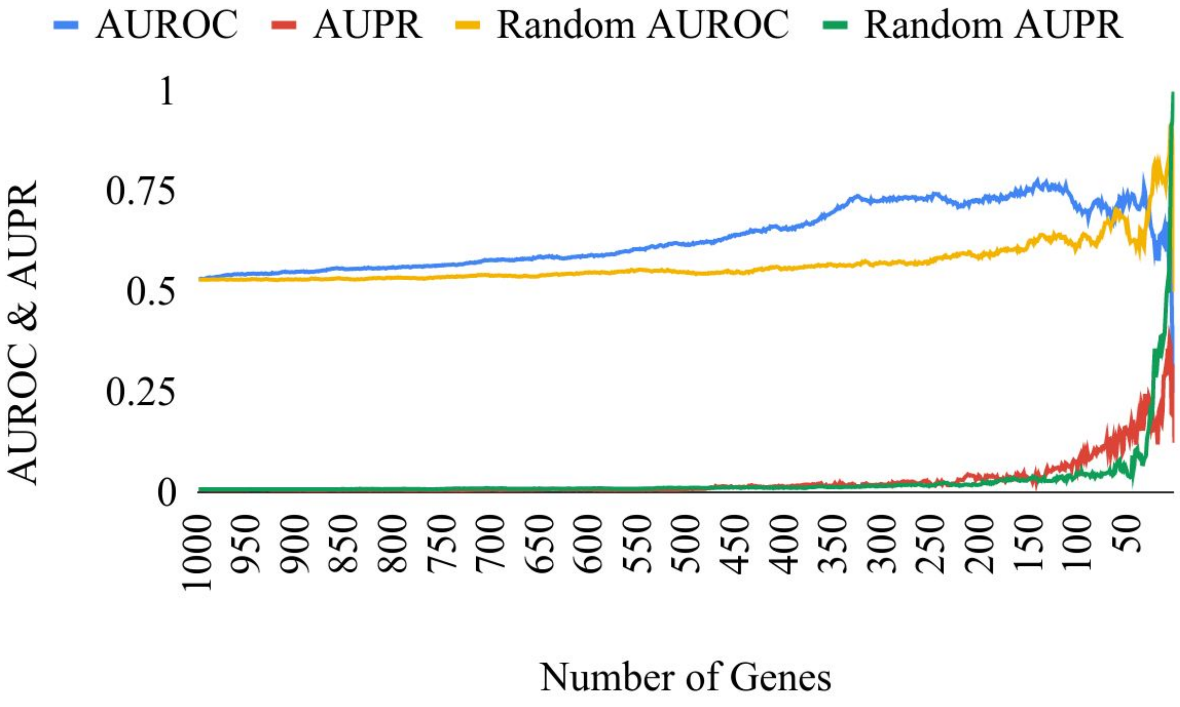

Supplementary Figure 3. Performances of both the gene reduction algorithm and random removal on a 1000-gene synthetic dataset. The x-axis represents the subset size, and the y-axis denotes the AUROC and AUPR values.

Supplementary Table 2. Cell line data set dimensions, SNR levels, and the number of empty vectors per cell line.

Cell Line	Size	SNR_L	# Empty
**MCF7**	512x1282	0.0030	37
**A375**	649x1879	0.0020	65
**HEPG2**	650x2129	0.0030	3
**VCAP***	664x1717	0.0021	56
**PC3**	697x1670	0.0023	81
**HA1E**	716x2092	0.0017	24
**A549**	744x2016	0.0017	30
**HT29**	746x2901	0.0012	33
**HCC515**	803x2390	0.0010	34

* VCAP expression values belong to 120H measurements while all the others belong to 96H.

Supplementary Table 3. Up and Down Regulation

	**Significantly**		**Insignificantly**	
**Cell Line**	**Down**	**Up**	**Down**	**Up**
**MCF7**	274	56	114	68
**A375**	489	33	73	54
**HEPG2**	382	24	187	57
**VCAP***	350	91	132	91
**PC3**	363	71	162	101
**HA1E**	441	115	79	81
**A549**	422	37	193	92
**HT29**	436	26	188	96
**HCC515**	423	46	224	110

* VCAP expression values belong to 120H measurements while all the others belong to 96H.

Knockdown effect on the target

Since a successful knockdown of the target gene is important, the knockdown effects on the targets were investigated. Volcano plots of the knockdown effects are provided in figures S1-18. Significance analyses of the targeted genes show that a majority of the targets were successfully downregulated. However, in addition to the insignificant changes in their expressions, a portion of the targets was observed to be significantly upregulated. In A375, A549, HCC515, HEPG2, and HT29, 6-10% of the genes were significantly upregulated, while this proportion increases up to 20-26% in HA1E, MCF7, PC3 and VCAP cell lines.

Fold changes are calculated based on the averages of these columns. P values are calculated by Mann Whitney-U test and corrected according to Benjamini-Hochberg (BH) test.

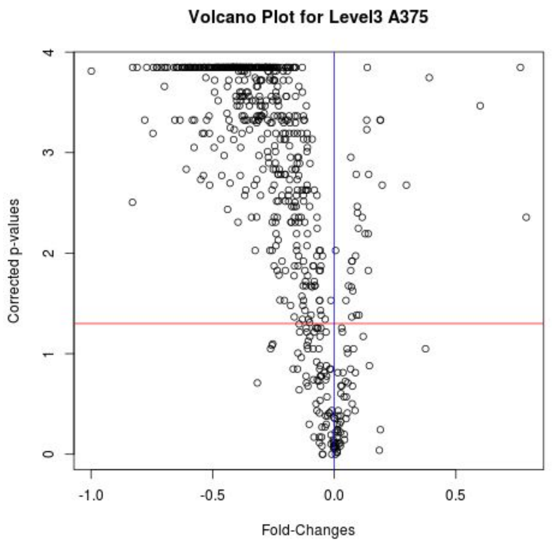

Supplementary Figure 4. The volcano plot showing the knockdown effect on A375 cell line targets from Mann-Whitney U test of the fold change target expression vs control.

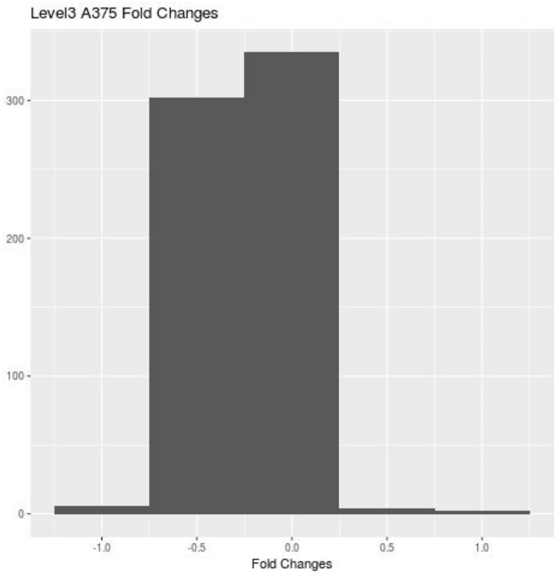

Supplementary Figure 5. The histogram of the fold change target expressions of A375 genes. The x- and y-axes show the fold changes and their frequencies, respectively.

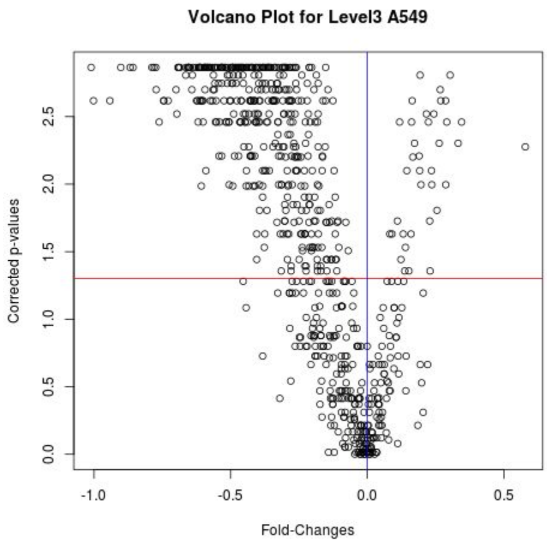

Supplementary Figure 6. The volcano plot showing the knockdown effect on A549 cell line targets from Mann-Whitney U test of the fold change target expression vs control.

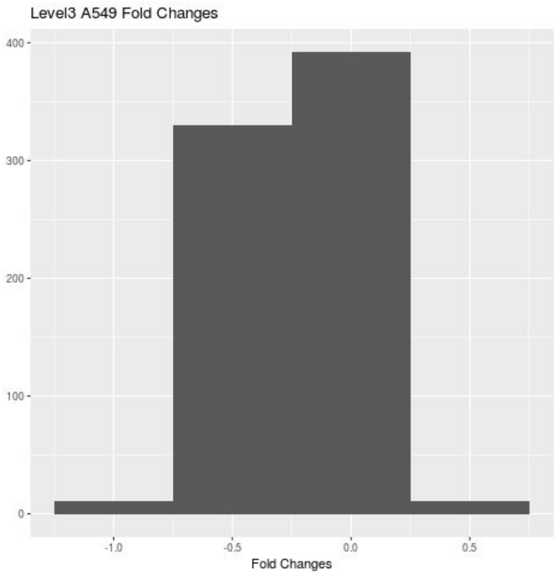

Supplementary Figure 7. The histogram of the fold change target expressions of A549 genes. The x- and y-axes show the fold changes and their frequencies, respectively.

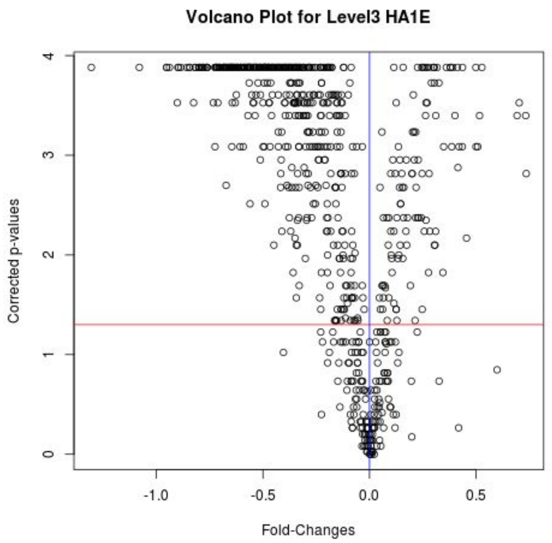

Supplementary Figure 8. The volcano plot showing the knockdown effect on HA1E cell line targets from Mann-Whitney U test of the fold change target expression vs control.

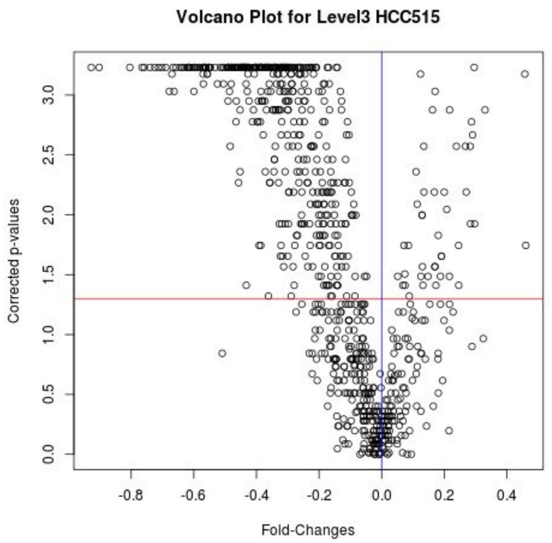

Supplementary Figure 10. The volcano plot showing the knockdown effect on HCC515 cell line targets from Mann-Whitney U test of the fold change target expression vs control.

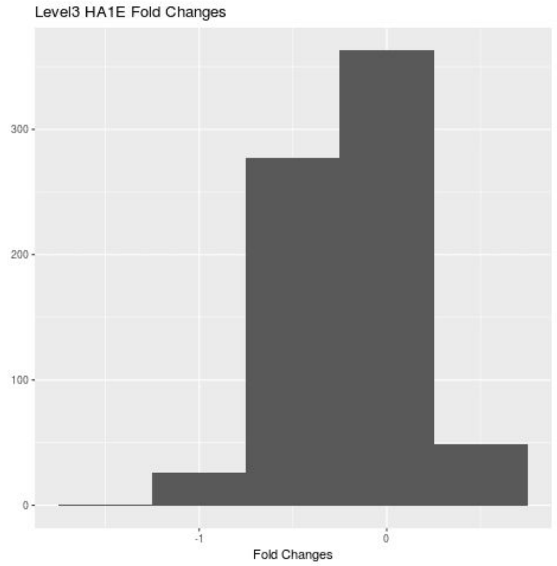

Supplementary Figure 9. The histogram of the fold change target expressions of HA1E genes. The x- and y-axes show the fold changes and their frequencies, respectively.

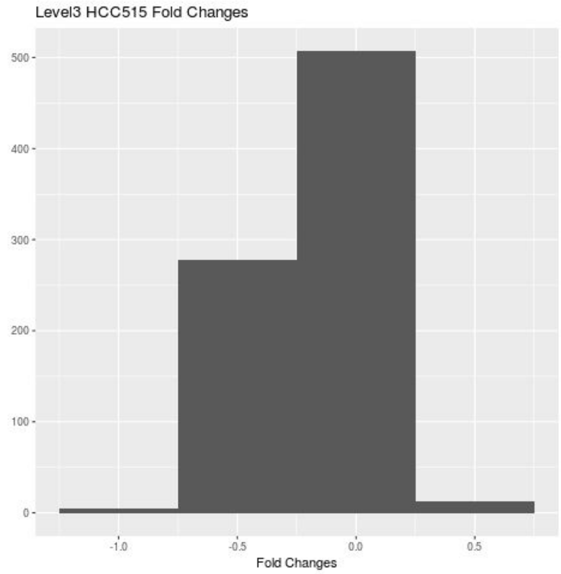

Supplementary Figure 11. The histogram of the fold change target expressions of HCC515 genes. The x- and y-axes show the fold changes and their frequencies, respectively.

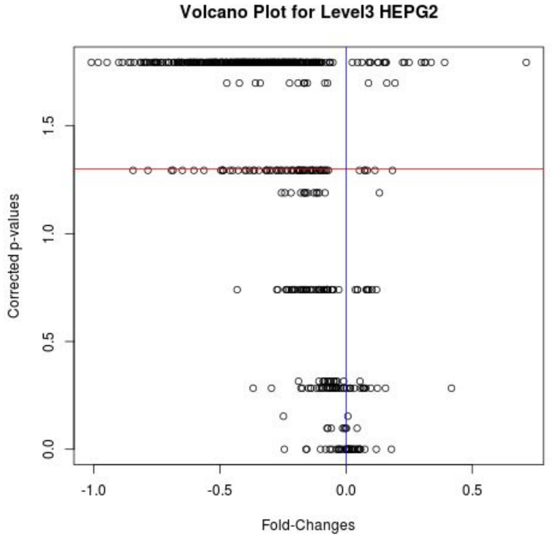

Supplementary Figure 12. The volcano plot showing the knockdown effect on HEPG2 cell line targets from Mann-Whitney U test of the fold change target expression vs control.

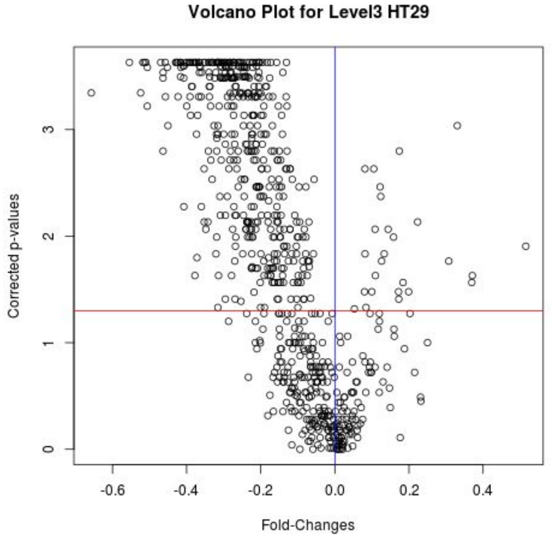

Supplementary Figure 14. The volcano plot showing the knockdown effect on HT29 cell line targets from Mann-Whitney U test of the fold change target expression vs control.

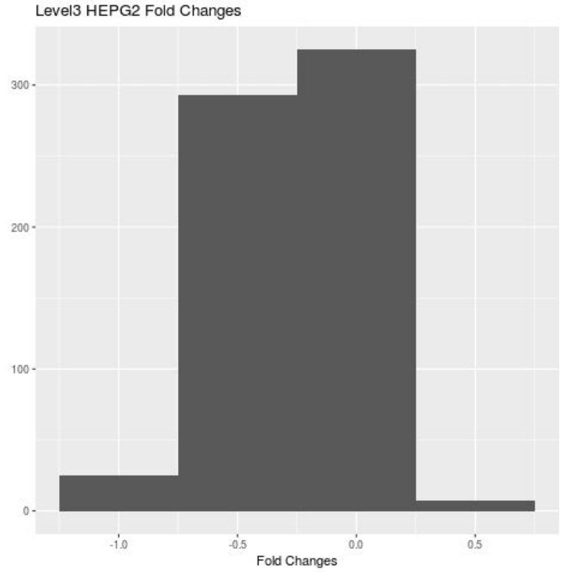

Supplementary Figure 13. The histogram of the fold change target expressions of HEPG2 genes. The x- and y-axes show the fold changes and their frequencies, respectively.

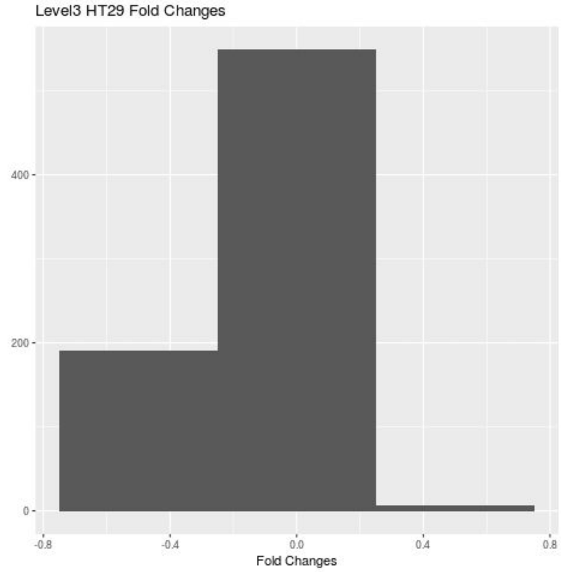

Supplementary Figure 15. The histogram of the fold change target expressions of HT29 genes. The x- and y-axes show the fold changes and their frequencies, respectively.

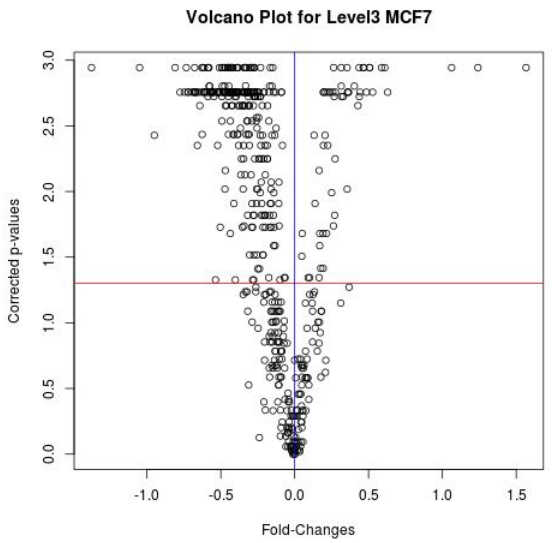

Supplementary Figure 16. The volcano plot showing the knockdown effect on MCF7 cell line targets from Mann-Whitney U test of the fold change target expression vs control.

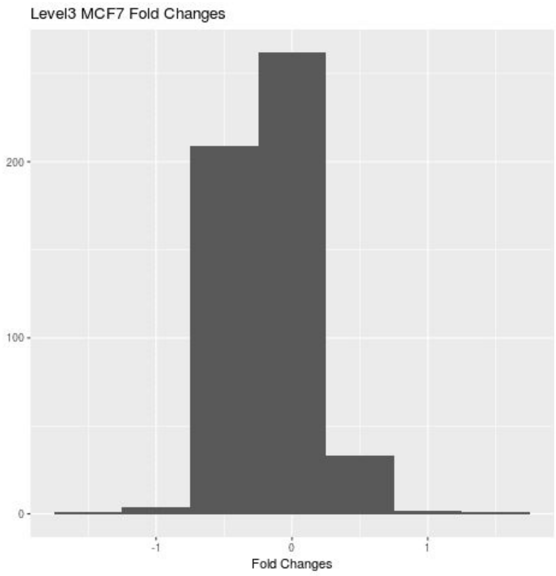

Supplementary Figure 17. The histogram of the fold change target expressions of MCF7 genes. The x- and y-axes show the fold changes and their frequencies, respectively.

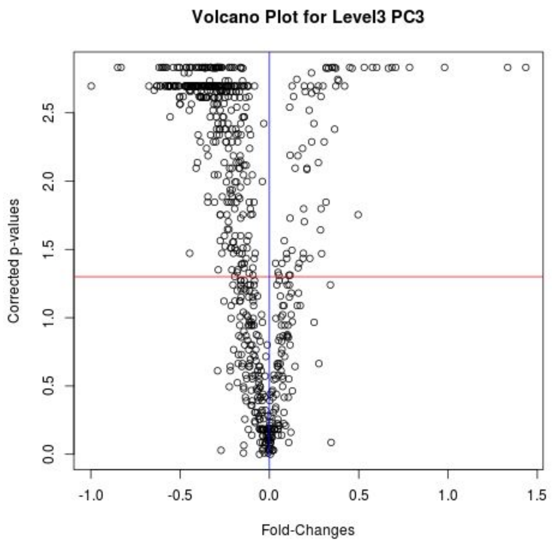

SupplementaryFigure 18. The volcano plot showing the knockdown effect on PC3 cell line targets from Mann-Whitney U test of the fold change target expression vs control.

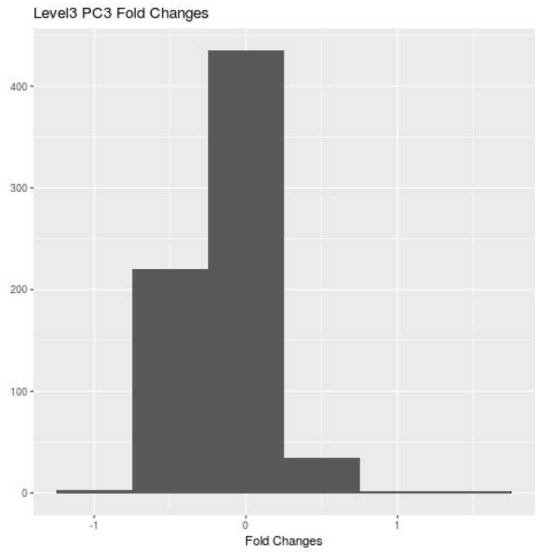

Supplementary Figure 19. The histogram of the fold change target expressions of PC3 genes. The x- and y-axes show the fold changes and their frequencies, respectively.

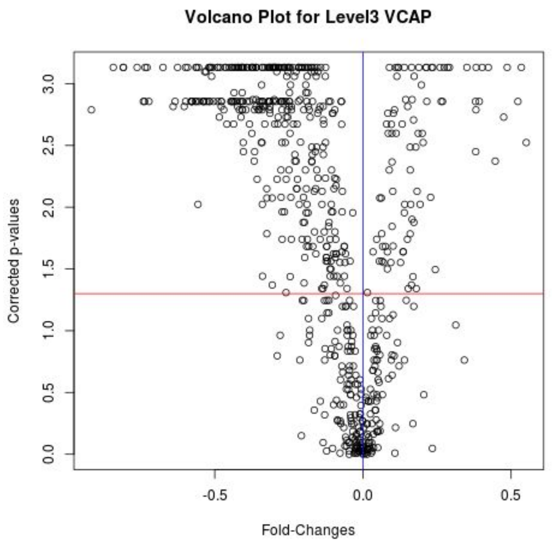

Supplementary Figure 20. The volcano plot showing the knockdown effect on VCAP cell line targets from Mann-Whitney U test of the fold change target expression vs control.

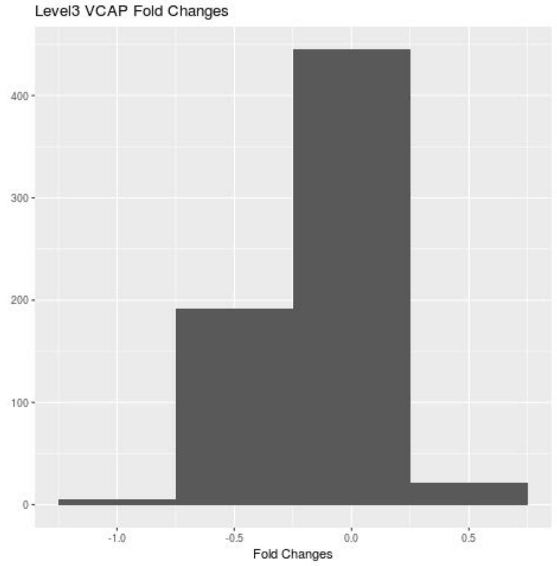

Supplementary Figure 21. The histogram of the fold change target expressions of VCAP genes. The x- and y-axes show the fold changes and their frequencies, respectively.

Benchmarking of the inference methods is performed for the nine L1000 cell lines with the possible combinations of LSCO and LASSO in the inference/generation of the true GRN and the inference of the GRNs from the subsets. To illustrate, LSCO&LSCO means that the generation of the true GRN is done with LSCO and the GRNs are further inferred also with LSCO from the L1000 subsets, whereas in LSCO&LASSO, the true network is generated with LSCO and the GRNs are inferred with LASSO from the subsets. Figues S19-36 show the comparison of the performances of these two inference methods in possible combinations in terms of AUROC and AUPR.

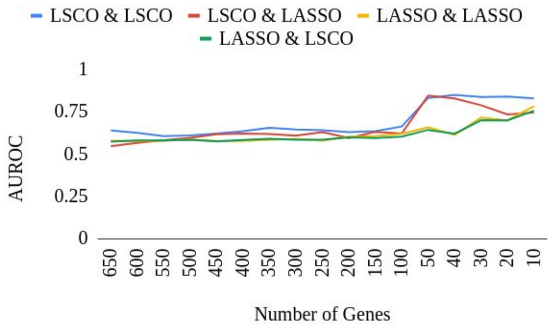

Supplementary Figure 22. Evaluation of methods for benchmarking GRN inference accuracy with simulation, for the subsets of the A375 cell line in terms of AUROC.

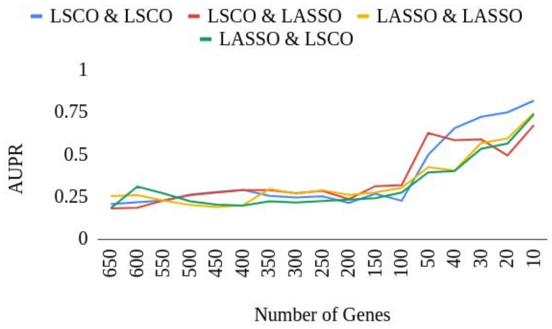

Supplementary Figure 23. Evaluation of methods for benchmarking GRN inference accuracy with simulation, for the subsets of the A375 cell line in terms of AUPR.

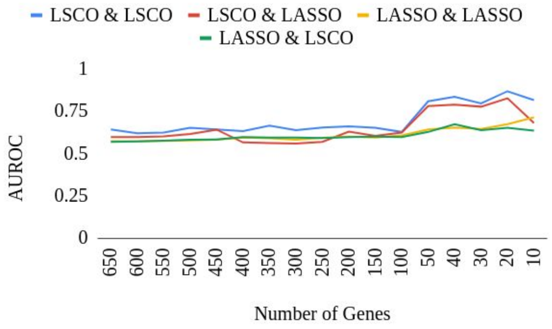

Supplementary Figure 24. Evaluation of methods for benchmarking GRN inference accuracy with simulation, for the subsets of the A549 cell line in terms of AUROC.

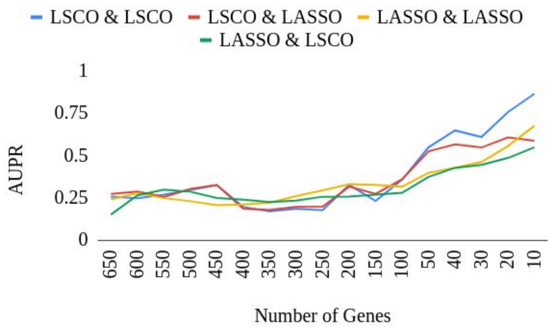

Supplementary Figure 25. Evaluation of methods for benchmarking GRN inference accuracy with simulation, for the subsets of the A549 cell line in terms of AUPR.

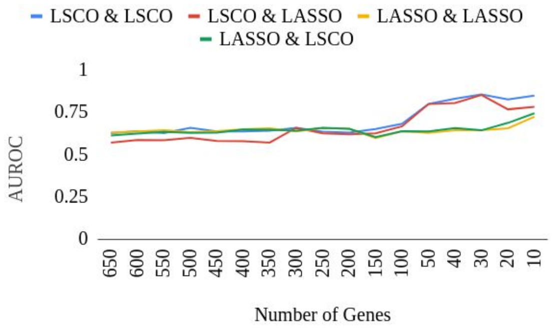

Supplementary Figure 26. Evaluation of methods for benchmarking GRN inference accuracy with simulation, for the subsets of the HA1E cell line in terms of AUROC.

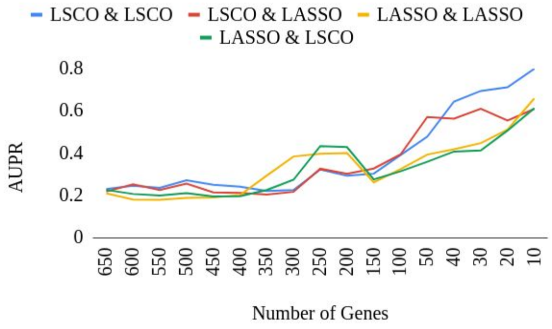

Supplementary Figure 27. Evaluation of methods for benchmarking GRN inference accuracy with simulation, for the subsets of the HA1E cell line in terms of AUPR.

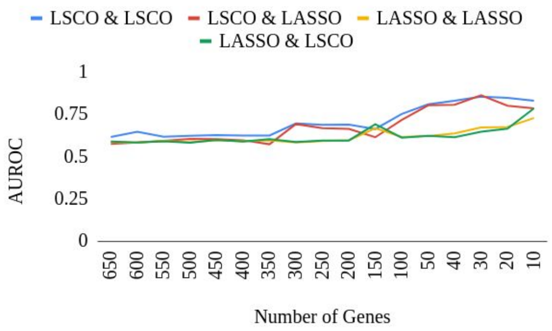

Supplementary Figure 28. Evaluation of methods for benchmarking GRN inference accuracy with simulation, for the subsets of the HCC515 cell line in terms of AUROC.

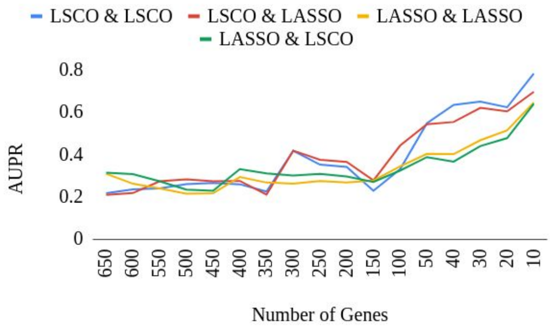

Supplementary Figure 29. Evaluation of methods for benchmarking GRN inference accuracy with simulation, for the subsets of the HCC515 cell line in terms of AUPR.

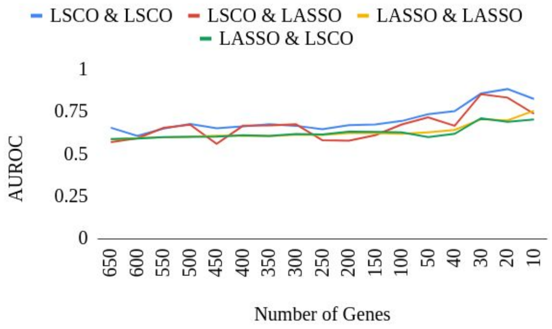

Supplementary Figure 30. Evaluation of methods for benchmarking GRN inference accuracy with simulation, for the subsets of the HEPG2 cell line in terms of AUROC.

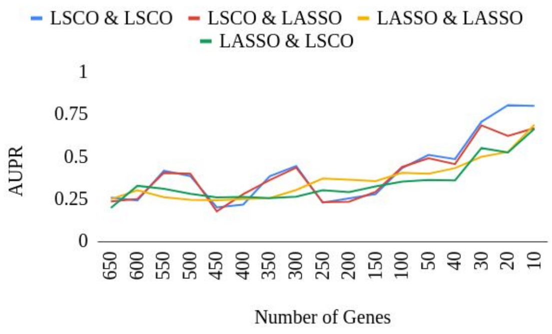

Supplementary Figure 31. Evaluation of methods for benchmarking GRN inference accuracy with simulation, for the subsets of the HEPG2 cell line in terms of AUPR.

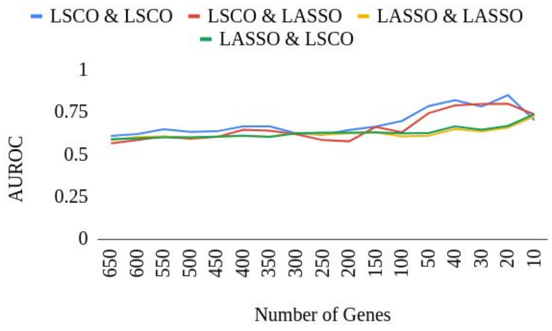

Supplementary Figure 32. Evaluation of methods for benchmarking GRN inference accuracy with simulation, for the subsets of the HT29 cell line in terms of AUROC.

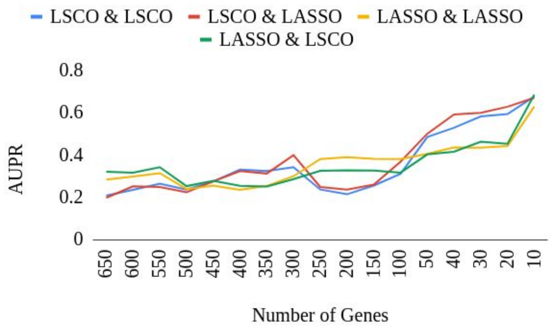

Supplementary Figure 33. Evaluation of methods for benchmarking GRN inference accuracy with simulation, for the subsets of the HT29 cell line in terms of AUPR.

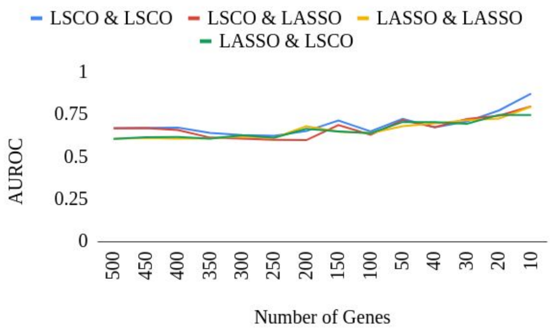

Supplementary Figure 34. Evaluation of methods for benchmarking GRN inference accuracy with simulation, for the subsets of the MCF7 cell line in terms of AUROC.

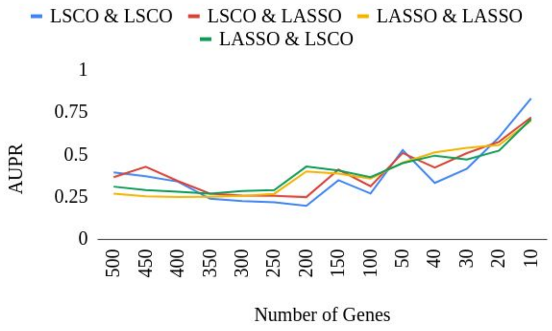

Supplementary Figure 35. Evaluation of methods for benchmarking GRN inference accuracy with simulation, for the subsets of the MCF7 cell line in terms of AUPR.

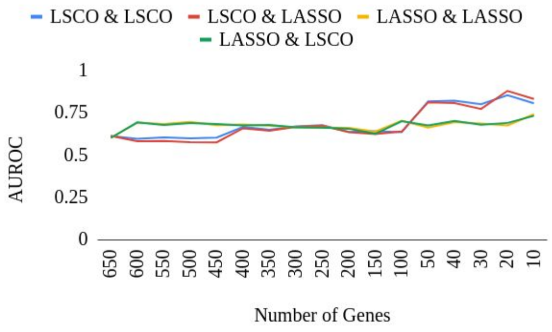

Supplementary Figure 36. Evaluation of methods for benchmarking GRN inference accuracy with simulation, for the subsets of the PC3 cell line in terms of AUROC.

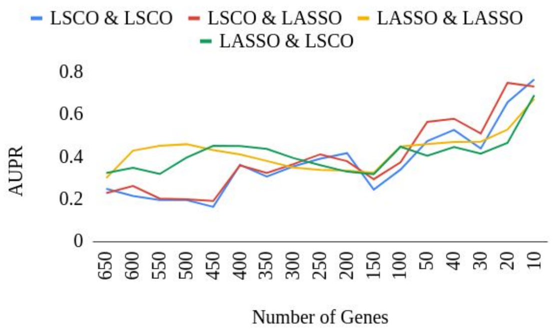

Supplementary Figure 37. Evaluation of methods for benchmarking GRN inference accuracy with simulation, for the subsets of the PC3 cell line in terms of AUPR.

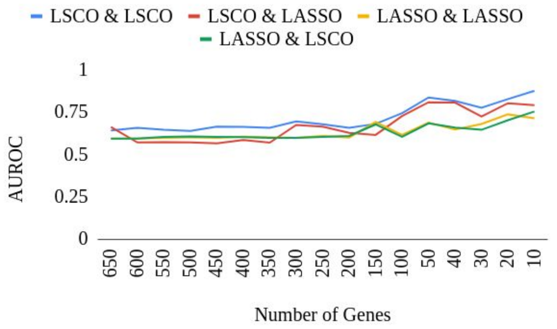

Supplementary Figure 38. Evaluation of methods for benchmarking GRN inference accuracy with simulation, for the subsets of the VCAP cell line in terms of AUROC.

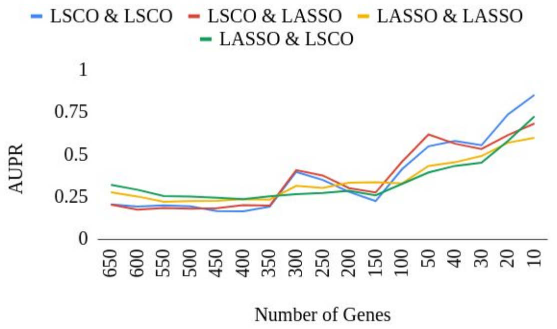

Supplementary Figure 39. Evaluation of methods for benchmarking GRN inference accuracy with simulation, for the subsets of the VCAP cell line in terms of AUPR.

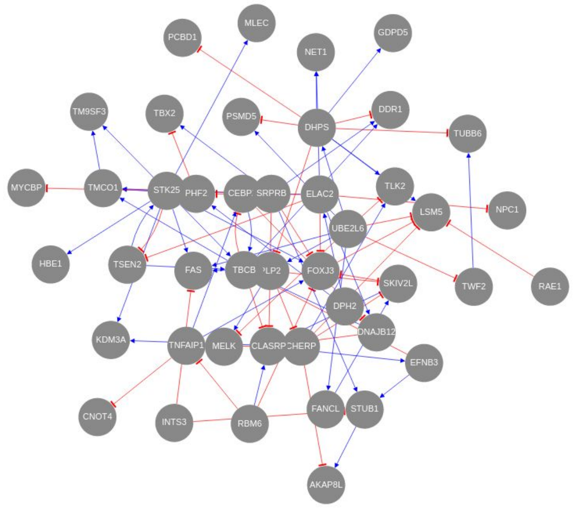

Supplementary Figure 40. The accurate GRN of the A375 cell line. The nodes demonstrate the informative genes of this L1000 cell line, and blue and red edges represent the regulatory interactions which are either activation or suppression, respectively.

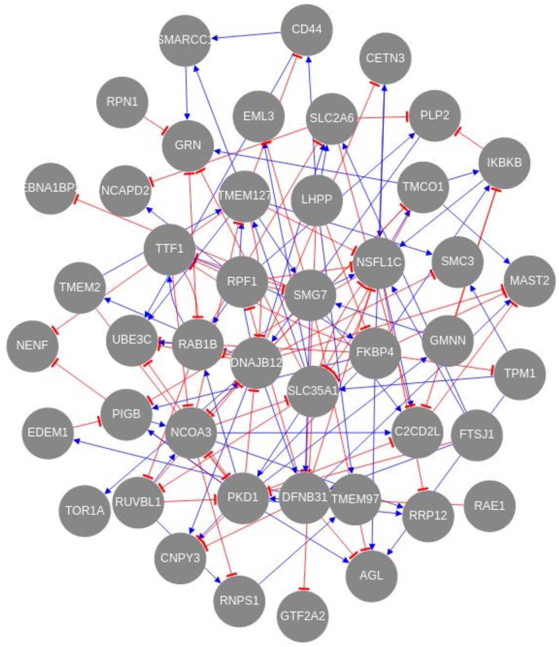

Supplementary Figure 41. The accurate GRN of the A549 cell line. The nodes demonstrate the informative genes of this L1000 cell line, and blue and red edges represent the regulatory interactions which are either activation or suppression, respectively.

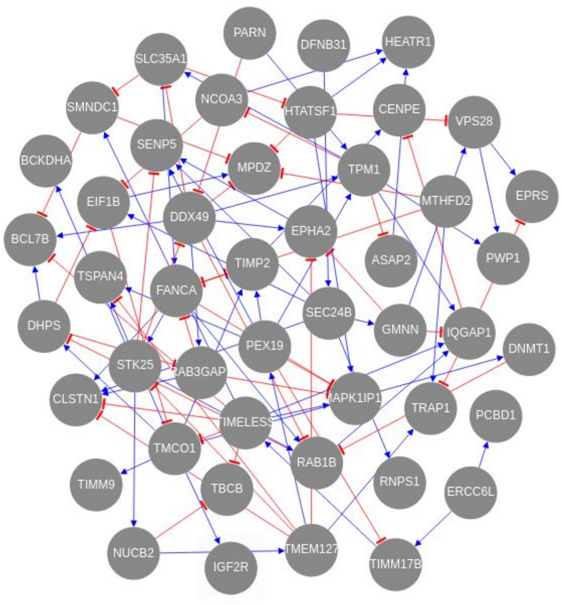

Supplementary Figure 42. The accurate GRN of the HA1E cell line. The nodes demonstrate the informative genes of this L1000 cell line, and blue and red edges represent the regulatory interactions which are either activation or suppression, respectively.

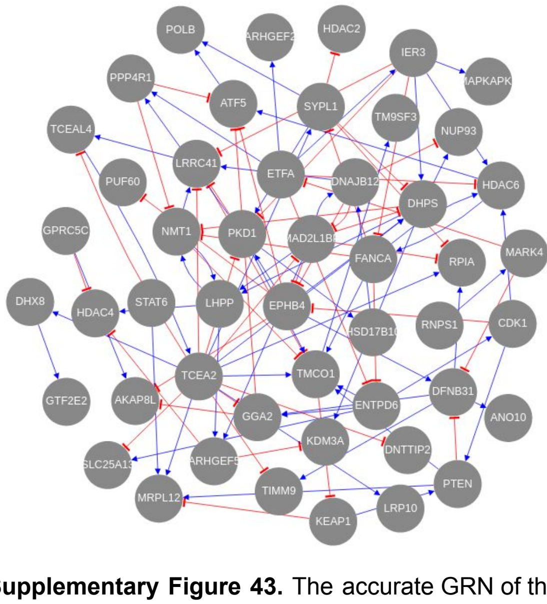

Supplementary Figure 43. The accurate GRN of the HCC515 cell line. The nodes demonstrate the informative genes of this L1000 cell line, and blue and red edges represent the regulatory interactions which are either activation or suppression, respectively.

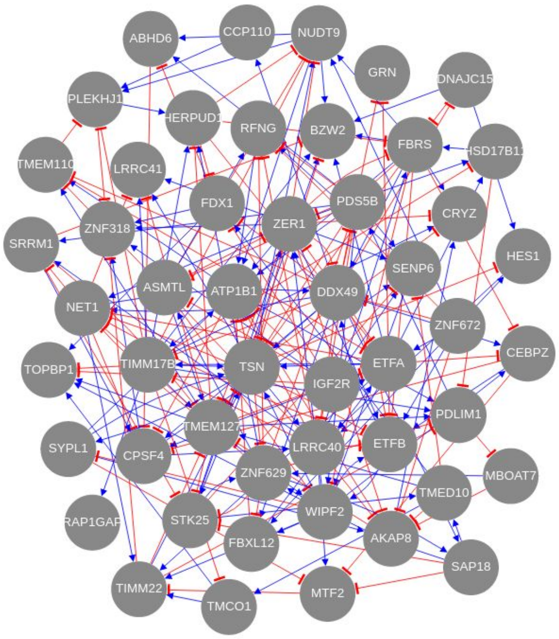

Supplementary Figure 44. The accurate GRN of the HEPG2 cell line. The nodes demonstrate the informative genes of this L1000 cell line, and blue and red edges represent the regulatory interactions which are either activation or suppression, respectively.

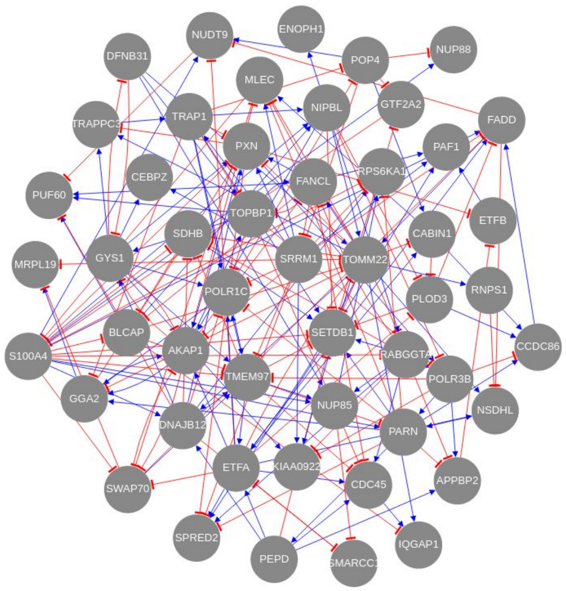

Supplementary Figure 45. The accurate GRN of the HT29 cell line. The nodes demonstrate the informative genes of this L1000 cell line, and blue and red edges represent the regulatory interactions which are either activation or suppression, respectively.

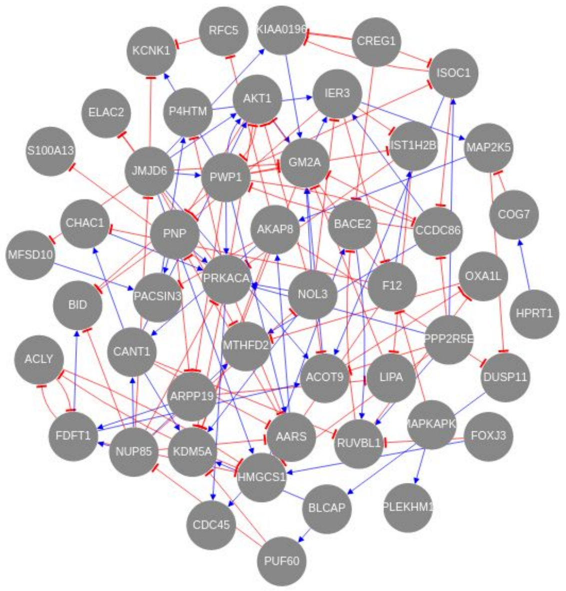

Supplementary Figure 46. The accurate GRN of the MCF7 cell line. The nodes demonstrate the informative genes of this L1000 cell line, and blue and red edges represent the regulatory interactions which are either activation or suppression, respectively.

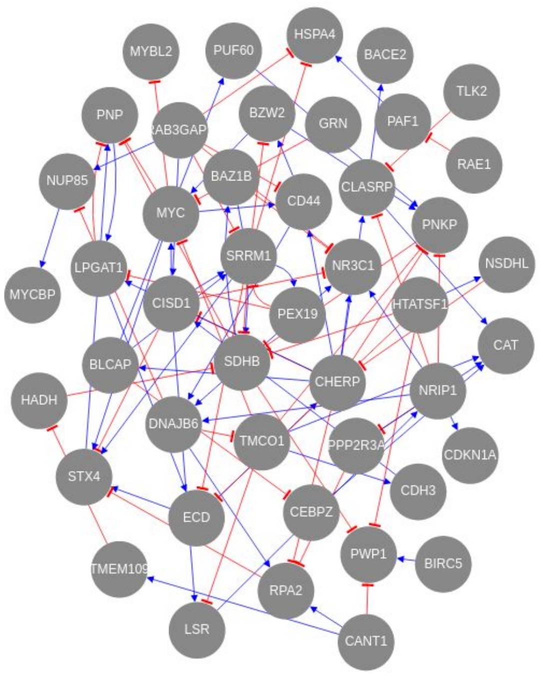

Supplementary Figure 47. The accurate GRN of the PC3 cell line. The nodes demonstrate the informative genes of this L1000 cell line, and blue and red edges represent the regulatory interactions which are either activation or suppression, respectively.

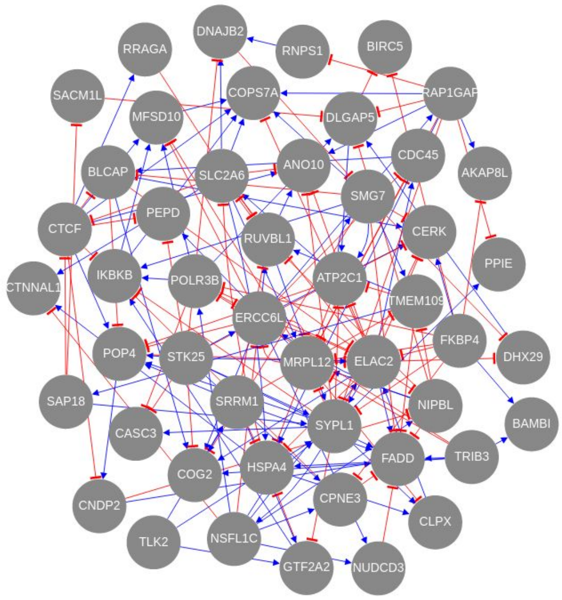

Supplementary Figure 48. The accurate GRN of the VCAP cell line. The nodes demonstrate the informative genes of this L1000 cell line, and blue and red edges represent the regulatory interactions which are either activation or suppression, respectively.

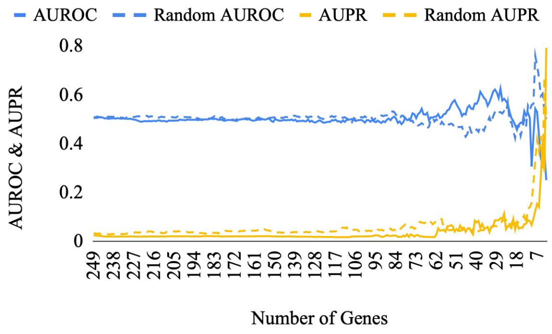

Supplementary Figure 49. Exploring the effectiveness of SNR 0.0001 for 250 genes and 750 experiments. The true GRN and the dataset was generated via GeneSPIDER.

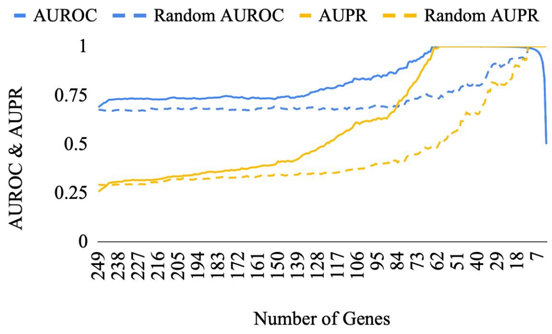

Supplementary Figure 50. Exploring the effectiveness of SNR 0.01 for 250 genes and 750 experiments. The true GRN and the dataset was generated via GeneSPIDER.

Entropy-based subset selection

We calculated the absolute gene expression for each gene by $\mathbf{2^{(fold\ change)}}$, and then calculated the gene entropy as in Zambelli et al. (2018).

The genes having high entropy values were considered as the ones not varying sufficiently. The entropy-based removal of genes was performed by removing the highest entropy gene until 2 genes remained.

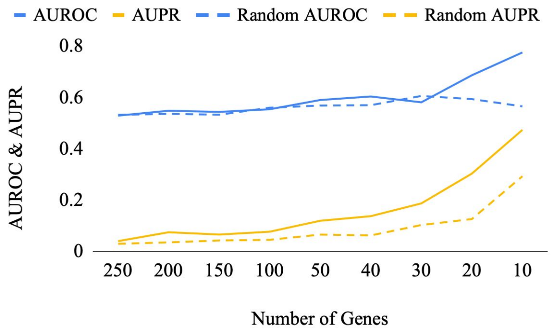

Supplementary Figure 51. Accuracy measures in terms of AUROC and AUPR from the entropy-based removal on the 250-gene dataset generated via GeneSPIDER.

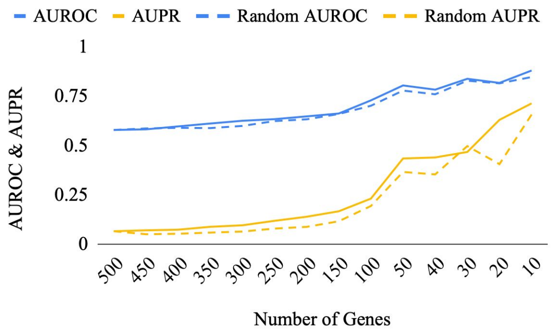

Supplementary Figure 52. Accuracy measures in terms of AUROC and AUPR from the entropy-based removal on the 500-gene dataset generated via GeneSPIDER.

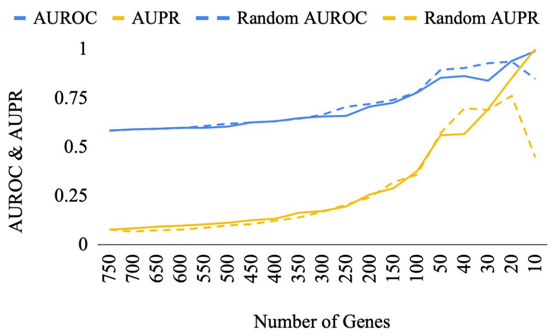

Supplementary Figure 53. Accuracy measures in terms of AUROC and AUPR from the entropy-based removal on the 750-gene dataset generated via GeneSPIDER.

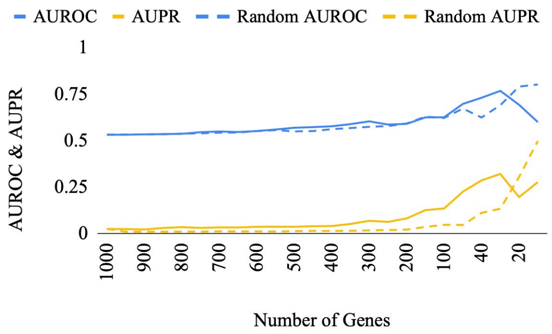

Supplementary Figure 54. Accuracy measures in terms of AUROC and AUPR from the entropy-based removal on the 1000-gene dataset generated via GeneSPIDER.

Supplementary Table 4. Calculation of the p-values from the hypergeometric test per subset cell line.

p = 1-phyper(overlapping links -1, links in db, non-link in db, links in GRN)
A375 <- 1-phyper(6-1, 36, 49*49-49-36, 84)
A549 <- 1-phyper(4-1, 106, 50*50-50-106, 130)
HA1E <- 1-phyper(6-1, 90, 50*50-50-90, 96)
HCC515 <- 1-phyper(6-1, 74, 50*50-50-74, 104)
HEPG2 <- 1-phyper(7-1, 33, 50*50-50-33, 221)
HT29 <- 1-phyper(13-1, 97, 50*50-50-97, 197)
MCF7 <- 1-phyper(19-1, 79, 50*50-50-79, 125)
PC3 <- 1-phyper(10-1, 77, 50*50-50-77, 91)
VCAP <- 1-phyper(10-1, 88, 50*50-50-88, 170)

Supplementary Table 5. The comparison of the full GRN of each cell line to the related FunCoup network of the same genes.

Cell Line	Overlap	Links in FunCoup	Links in the inferred GRN	p-value
A375	170	23776	2458	0.933
A549	182	31947	3038	0.999
HA1E	63	30516	1018	0.992
HCC515	120	39855	1521	0.756
HEPG2	155	24152	2289	0.974
HT29	275	34105	3115	0.137
MCF7	112	15180	1825	0.998
PC3	116	28416	1497	0.599
VCAP	183	26863	2718	0.999

Supplementary Table 6. PathwAX results for the significant association of the subnetwork genes to KEGG pathways.

Cell Line	Associated Pathway	p-values
A375	N-Glycan biosynthesis	7.75e-6
	Protein processing in ER	4.72e-3
	ABC transporters	6.76e-3
	Glycerophospholipid metabolism	1.54e-2
A549	Glycosylphosphotidylinositol (GPI)-anchor biosynthesis	3.92e-4
	Cell cycle	5.07e-3
	Steroid biosynthesis	3.29e-2
HA1E	-	
HCC515	Hepatitis B	7.48e-3
	Non-alcoholic fatty liver disease (NAFLD)	1.19e-2
	Valine, leucine and isoleucine degradation	4.05e-2
	Cell cycle	4.77e-2
HEPG2	Alzheimer’s disease	4.59e-21
	Oxidative phosphorylation	9.98e-19
	Parkinson’s disease	1.82e-16
	Valine, leucine and isoleucine degradation	1.91e-15
	Non-alcoholic fatty liver disease (NAFLD)	1.00e-13
	Huntington’s disease	3.89e-12
	Fatty acid degradation	1.11e-10
	Propanoate metabolism	2.76e-4
	Beta-alanine metabolism	7.18e-4
	Cardiac muscle contraction	9.22e-4
	Lysosome	1.19e-3
	Lysine degradation	6.24e-3
	Butanoate metabolism	1.15e-2
	Protein processing in ER	2.06e-2
HT29	Alzheimer’s disease	6.17e-12
	Parkinson’s disease	7.04e-10
	Non-alcoholic fatty liver disease (NAFLD)	1.03e-9
	Oxidative phosphorylation	1.41e-7
	Huntington’s disease	4.30e-7
	RNA transport	2.46e-3
	Valine, leucine and isoleucine degradation	4.50e-3
	Fatty acid degradation	4.42e-2
MCF7	-	
PC3	Peroxisome	6.12e-3
VCAP	Cell cycle	2.13e-4
	mRNA surveillance pathway	7.33e-3
	RNA transport	2.72e-2
